# Vitamin a potentiates sheep myoblasts myogenic differentiation through BHLHE40-modulated *ID3* expression

**DOI:** 10.1186/s12864-024-10161-0

**Published:** 2024-03-05

**Authors:** Pengkang Song, Jiamin Zhao, Weipeng Zhang, Xuying Li, Bingzhen Ji, Junxing Zhao

**Affiliations:** 1 College of Animal Science, Shanxi Agricultural University, 030801 Taigu, Shanxi P. R. China; 2 Shanxi Key Laboratory of Animal Genetics Resource Utilization and Breeding, Taigu, P. R. China

**Keywords:** Vitamin A, Lamb, Myoblast, BHLHE40, DNA binding inhibitor 3

## Abstract

**Background:**

Vitamin A and retinoic acid (RA, a metabolite of vitamin A), are inextricably involved to the development of skeletal muscle in animals. However, the mechanisms regulating skeletal muscle development by vitamin A remain poorly reported. The current study designed to investigate the underlying mechanism of vitamin A affecting myogenic differentiation of lamb myoblasts through transcriptome sequencing (RNA-Seq) and gene function validation experiments. It provides a theoretical basis for elucidating the regulation of vitamin A on skeletal muscle development as well as for improving the economic benefits of the mutton sheep industry.

**Results:**

Newborn lambs were injected with 7,500 IU vitamin A, and *longissimus dorsi* (LD) muscle tissue was surgically sampled for RNA-Seq analysis and primary myoblasts isolation at 3 weeks of age. The results showed that a total of 14 down-regulated and 3 up-regulated genes, were identified between control and vitamin A groups. Among them, *BHLHE40* expression was upregulated in vitamin A group lambs. Furthermore, *BHLHE40* expression is significantly increased after initiation of differentiation in myoblasts, and RA addition during differentiation greatly promoted *BHLHE40* mRNA expression. In vitro, RA inhibited myoblasts proliferation and promoted myoblasts myogenic differentiation through *BHLHE40*. Moreover, BHLHE40 was proved to inhibit the expression of the *DNA binding inhibitor 3* (*ID3*), and meanwhile, *ID3* could effectively promote myoblasts proliferation and inhibit myoblasts myogenic differentiation.

**Conclusions:**

Taken together, our results suggested that vitamin A inhibited myoblasts proliferation and promoted myoblasts myogenic differentiation by inhibiting *ID3* expression through BHLHE40.

**Supplementary Information:**

The online version contains supplementary material available at 10.1186/s12864-024-10161-0.

## Introduction

Skeletal muscle consists of multinucleated muscle fibers formed by the fusion of myoblasts, and accounts for approximately 40% of total body weight. Skeletal muscle performs numerous biological functions, including voluntary movement, controlling body temperature and regulating metabolism [1, 2]. Meanwhile, skeletal muscle, as the main source of animal protein, seriously affects the meat yield of farm animals at harvest [3, 4]. During myogenesis, myoblasts derived from the somatic mesoderm undergo a highly coordinated process of myogenic differentiation, culminating in the formation of mature skeletal muscle [5] and this event requires the concerted action of numerous transcription factors and signaling pathways [6, 7]. Hence, elucidating the underlying mechanisms of myoblast differentiation is critical for understanding of skeletal muscle development.

Class E basic helix-loop-helix protein 40 (BHLHE40, Aliases: BHLHB2/STRA13/DEC1/Sharp2) is a transcriptional repressor expressed in different cells and is mainly involved in the control of circadian rhythms and cell differentiation. It has been shown that the expression level of *BHLHE40* is related to cell growth arrest [8]. For myogenesis, the absence of *BHLHE40* leads to muscle necrosis in mice [9]. *ID3* is a member of the HLH superfamily and it lacks the basic DNA-binding structural domain and represses transcription by forming non-functional dimers that are unable to bind to DNA [10]. Thus, *ID3* is widely recognized as a negative regulator of the differentiation process. Previous studies suggest that *ID3* is negatively involved in myogenesis by inhibiting the activation of myogenic regulatory factors during myoblast differentiation [10–12].

Vitamin A is essential for growth and development in humans and animals. RA, the most important metabolite of vitamin A, performs the vast majority of vitamin A metabolic functions. After entering the nucleus, RA regulates the expression of target genes by binding to RA receptors [13]. Studies have shown that RA promotes myogenesis in zebrafish [14] and Angus cattle [15], while inhibit differentiation of chick embryo limb myoblasts [16]. Our previous publication indicated that intramuscular injection of vitamin A increased final body weight and loin eye area of lambs, and RA promoted sheep primary myoblasts early differentiation in vitro [17]. In the current study, we continue to investigate the mechanism by which vitamin A affect sheep skeletal muscle growth.

## Materials and methods

### Animal treatment and sample collection

A total of 80 purebred healthy Hu sheep were randomly selected, and all ewes were on their third pregnancy. To avoid the influence of sire, we used semen from one Dorper ram for artificial insemination. Every 3 ewes were placed in a shed and the ewes were fed a diet that met the National Research Council nutritional requirements for ewes. Ewes were exposed to consistent environmental conditions and had free access to clean water and salt blocks. The number of fetuses carried by the ewes was observed at day 35 of gestation using an ultrasonic detector. Only ewes with 2 fetuses were used for further experimentation. After the birth of lambs, male twins weighing 3.5 ± 0.5 Kg were selected and randomly assigned to control and treatment groups of 8 rams each.

Based on the previous studies’ recommendation [15, 18], 7500 IU vitamin A palmitate (vitamin A group, product no. PHR1235, Sigma) or an equivalent volume of corn oil (Control group, product no. c8267, Sigma) were injected into the biceps femoris muscle of 2-day-old lambs. After that, lambs in control and vitamin A group were injected once a week at a fixed time point for 3 weeks. An appropriate amount of LD muscle samples of 3-week-old lambs were surgically obtained for subsequent experiments, then the wound was cleaned and the skin was sutured.

### Transcriptome sequencing analysis (RNA-seq)

Total RNA in muscle tissue was extracted using a Trizol kit (Invitrogen, Carlsbad, CA, USA) and assessed for RNA quality. Eukaryotic mRNA was enriched using Oligo (dT) beads and the enriched mRNA was fragmented using fragmentation buffer and then reverse transcribed into cDNA. The obtained double-stranded cDNA was purified and end-repaired and ligated to the Illumina sequencing adapter for polymerase chain reaction (PCR) amplification. The obtained cDNA library was sequenced using Illumina Novaseq6000 (Gene Denovo Biotechnology Co., Guangzhou, China). The resulting reads were filtered by fastp [19] (version 0.18.0) to obtain high-quality, clean reads. The rRNA mapping reads were removed using Bowtie2 [20] (version 2.2.8). Then, mapping of paired-end clean reads to the reference genome was performed using HISAT2. 2.4 [21]. Using a reference-based method, the number of mapped reads for each sample was assembled using StringTie v1.3.1 [22, 23]. The FPKM (Fragments per kilobase of transcript per million mapped reads) values were calculated for each transcribed region using the RSEM [24] software to quantify its expression abundance and variation.

### Principal component analysis (PCA)

Principal component analysis (PCA) was performed with R package gmodels (http://www.r-project.org/) in this experiment. PCA is the degree of interpretation, which is largely used to reveal the structure/relationship of the samples/datas, and usually results are considered reliable with PCA greater than 50%.

### Differentially expressed gene (DEG) analysis

RNAs between two different groups were analyzed for differential expression using DESeq2 [25] software (and between two samples with edgeR) [26]. Genes/transcripts with a false discovery rate (FDR) parameter below 0.05 and an absolute fold change of ≥ 2 were considered as differentially expressed genes/transcripts. Differentially expressed genes are shown in additional file 1.

### Target gene prediction for BHLHE40

The target gene prediction of BHLHE40 was performed using online databases. First, three online databases, (GTRD, Cistrome Data Browser and hTFtarget) were used to predict the target genes of BHLHE40, and then the intersection of the three databases was taken to be the predicted target genes of BHLHE40. Finally, the target genes with stable binding sites to BHLHE40 and involved in the proliferation and differentiation of skeletal muscle cells were screened by reviewing the references and using the JASPAR online database.

### Isolation, culture and treatment of primary myoblasts

Surgically obtained skeletal muscle tissue was washed three times with PBS, and then the membrane, fat, and other surrounding non-muscle tissues were removed. The muscle was minced with scissors and placed in a 15 mL centrifuge tube, and digested with 0.1% collagenase type I for 45 min on a shaker at 37 °C, then centrifuged at 500 × g for 5 min, and the precipitate was retained. Using Dulbecco’s Modified Eagle’s medium (DMEM) resuspended the muscle pellet, and passed it sequentially through 100 μm and 40 μm cell strainers, then the filtrate was collected and centrifuged at 500 × g for 5 min. Finally, the cell pellet was resuspended with DMEM containing 20% fetal bovine serum (FBS), 0.1% penicillin, and 0.1% streptomycin and cultured in an incubator at 37 °C and 5% CO2. For studies of primary myoblasts differentiation, the medium containing 20% FBS was replaced with differentiation medium containing 2% horse serum. Based on previous study [27], the retinoic acid concentration in subsequent cell experiments was 100 nM.

### Transfection

In order to verify the function of BHLHE40 on the proliferation and differentiation of myoblasts, we transfected the cells with si-BHLHE40 and BHLHE40 overexpression vectors (Genomeditech, Shanghai, China) by using lipofectamine 3000, to inhibit and overexpress BHLHE40, respectively. Negative control siRNA (si-NC) and si-BHLHE40 sequences were shown in Table 1. To further investigate the effect of *ID3* (target gene of BHLHE40) on the proliferation and differentiation of myoblasts, we transfected the cells with si-ID3 and ID3 overexpression vectors (Genomeditech, Shanghai, China) using lipofectamine 3000. Negative control siRNA (si-NC) and si-ID3 sequences were shown in Table 2. Overexpression vector information for *BHLHE40* and *ID3* are shown in additional file 2.

**Table 1:** Si-NC and si-BHLHE40 sequences for cell transfection

Item	Sequence $(5^{\prime}{\rightarrow}3^{\prime})$
si-NC	UUCUCCGAACGUGUCACGUTT ACGUGACACGUUCGGAGAATT
si-BHLHE40	GGAGAAAGGAUCAGUGCUATT UAGCACUGAUCCUUUCUCCTT

**Table 2:** Si-NC and si-ID3 sequences for cell transfection

Item	Sequence $(5^{\prime}{\rightarrow}3^{\prime})$
si-NC	UUCUCCGAACGUGUCACGUTT ACGUGACACGUUCGGAGAATT
si-ID3	CAACCUCAUUGCUCAGUAUTT AUACUGAGCAAUGAGGUUGTT

### Wound healing scratch assay

Sheep primary myoblasts were seeded into 12-well plates to form a fused monolayer in incubator at 37 °C and 5% CO2. Then, a sterile 200 µL pipette tip was used to leave a scratch, and the sheep primary myoblasts were washed with PBS. For the *BHLHE40* silencing assay, myoblasts were treated with si-NC, si-NC + RA, si-BHLHE40 and si-BHLHE40 + RA, and for the *BHLHE40* overexpression assay, myoblasts were treated with empty vector and *BHLHE40* overexpression vector. Moreover, for the *ID3* silencing assay, myoblasts were treated with si-NC and si-ID3, and for the *ID3* overexpression assay, myoblasts were treated with empty vector and *ID3* overexpression vector. Three replicate wells for each treatment in the above test, and using a DMi8 microscope (Leica, Germany) to monitor cell migration at 0, 12 and 24 h after scratch formation.

### 5-ethynyl-2’-deoxyuridine staining assay (EdU)

EdU staining assays were performed using the Cell-Light EdU DNA Cell Proliferation Kit (RiboBio, Guangzhou, China). Specifically, sheep primary myoblasts were seeded into 12-well plates in incubator at 37 °C and 5% CO2, and the cells were treated exactly as in the scratch assay. Sheep primary myoblasts in logarithmic growth phase were treated for 24 h, and then cultured with EdU medium for 2 h and washed twice with PBS. Paraformaldehyde (4%) was used to fix the sheep primary myoblasts for 30 min and washed twice with PBS. After that, sheep primary myoblasts were incubated with 0.5% TritonX-100 for 10 min, washed with PBS, and incubated with Apollo stain for 10 min. Finally, DNA was stained with Hoechst 33,342 for 30 min and images were captured with a DMi8 microscope (Leica, Germany).

### Immunocytochemical staining

Sheep primary myoblasts were cultured to 100% fusion and induced to form myotubes, and paraformaldehyde (4%) was used to fix the cells on ice for 10 min. Using 0.25% Triton X-100 permeabilized myoblasts for 10 min and blocked with 1% bovine serum albumin for 1.5 h. Primary antibody (MHC) was used to incubate myoblasts overnight at 4 °C, and then corresponding secondary antibody was used to incubate myoblasts for 1 h at room temperature. Finally, DNA was stained with DAPI for 3 min and images were acquired under a DMi8 microscope (Leica, Germany). Finally, DNA was stained with DAPI for 3 min and images were acquired under a DMi8 microscope (Leica, Germany).

### Dual-luciferase reporter assay

Detailed information on plasmid construction of sheep ID3 promoter reporter gene is provided in additional file 3. When the cell coverage reached 70%, the medium was replaced with fresh medium. The sea cucumber endogenous plasmid and promoter reporter gene plasmid were transfected into the target cells 2 h later. After 48 h, the cell samples were collected, and the assay was performed according to the instructions of the reporter gene assay kit (Genomeditech, Shanghai, China). Briefly, the collected cell samples were sufficiently lysed, centrifuged at 1000 × g for 5 min, and the supernatant was retained. The Renilla Luciferase Assay working solution was prepared at room temperature. Finally, the parameters were set and analyzed using a full-featured microplate detector.

### Real-time PCR (qRT-PCR)

TRIzol reagent (Takara, Dalian, China) was used to extract total RNA on muscle and cells. Then, using a one-step reverse transcription kit (Takara, Dalian, China) to reverse transcribe the RNA to cDNA, and further analyzed for quantitative analysis of the target genes using a SYBR Green RT-PCR kit (TAKARA Co, Ltd) and a CFX RT-PCR detection system (Bio-Rad, Hercules, CA). The primer sequences for the target genes are shown in Table 3, and at least three replicates were performed for each sample. The data obtained in the experiments were analyzed for the relative expression of the target genes using the 2^−ΔΔCt^ method with β-actin as an internal reference gene.

**Table 3:** Primer sequences for Real-time PCR

Gene name	Sequence $(5^{\prime}{\rightarrow}3^{\prime})$	Product size, bp
*PITX3*	CATACAATGACCGCCCACTCT AGGCCTTCTCCGAATCGCT	240
*ABRA*	ACCAGAGGCTGGAAAGCGAT TCTCTGTGAATCTGTTTGCCTGGG	178
*ANKRD1*	AGCCCAGATCGAATTCCGTG GCGGTGCTGAGCAACTTATC	138
*SLC16A9*	CTTGCGAGTCTCGGATGTGG AAAGTCCAACACTTGAACCTGT	121
*BHLHE40*	GGAGACCTACCAGGGATGGAT TGCACTCGTTAATCCGGTCA	148
*MYF5*	CCCACCAGCCCCACCTCAAGT GTAGACGCTGTCAAAACTGCTGCT	93
*MYOD*	GAACTGCTACGACCGCACTTACT GAGATGCGCTCCACGATGCT	111
*MYOG*	CTCAACCAGGAGGAGCGCGAC TTGGGGCCAAACTCCAGTGCG	131
*ID3*	CGCATCTTCCCATCCAGACAG TTTGGGGAAGTCAAGTGGGC	206
*β-actin*	CGGCTTTCGGTTGAGCTGAC GCCGTACCCACCAGAGTGAA	159

### Western blotting

Protein lysis buffer (1% NaF, 1% Na3VO4, 1% PMSF, 2% β-mercaptoethanol, 0.1% protease inhibitor, 1 × loading buffer) was added to the cell samples, and the mixture was placed on ice for 10 min. Lysed cells were collected in 1.5 mL EP tubes, and the extracted proteins were denatured in boiling water for 10 min at 100 °C. Then, centrifugation was performed at 12,000 × g for 5 min, and the supernatant was collected and placed on ice. The soluble proteins were separated by SDS-PAGE at room temperature (80 V, 0.5 h, 120 V, 1.5 h) and then transferred to nitrocellulose membrane (100 V, 1.5 h). Skim milk (5%) was used to block the nitrocellulose membrane for 1 h at room temperature, and then incubated with primary antibody to the target protein overnight at 4 °C, and corresponding secondary antibody was incubated for 1 h at room temperature. Finally, the target proteins were visualized with an Odyssey infrared imaging system (LI-COR Biosciences, Lincoln, Nevada, USA) and the band densities were normalized to the β-actin content. (The blots were cut prior to hybridisation with antibodies, and images of blots for all replicates were provided in the additional file 4)

Antibodies against CDK4 (bs-0633R), Cyclin D1 (bs-0623R), MyoD (bs-23809R), MyoG (bs-3550), MHC (bs-18070R) and β-actin (bs-0061R) were purchased from Biosynthesis Biotechnology Co., Ltd. (Beijing, China). PCNA (#2586 s) was purchased from Cell Signaling (Danvers, MA, USA). Goat anti-rabbit secondary antibody (926 − 32,211) was from LI-COR Biosciences (Lincoln, NE, US), and anti-mouse fluoresce secondary antibodies (#4408) was from Cell Signaling (Danvers, MA, USA). ID3 (#DF12520) was from Affinity Biosciences (Jiangsu, China).

### Statistical analysis

All data in this experiment were processed using GraphPad Prism 9 software (Monrovia, CA, USA). The normal distribution and homogeneity of variance analysis were conducted. The comparison of two groups of data was performed using student’s *t*-test, and the comparison of multiple groups of data was performed using one-way analysis of variance (ANOVA) followed by Tukey’s test. Data were shown as the mean ± SEM. *P* < 0.05 was considered to be significantly different.

## Results

### Vitamin a promotes the *BHLHE40* expression in muscle and differentiated myoblasts

**Fig. 1:**
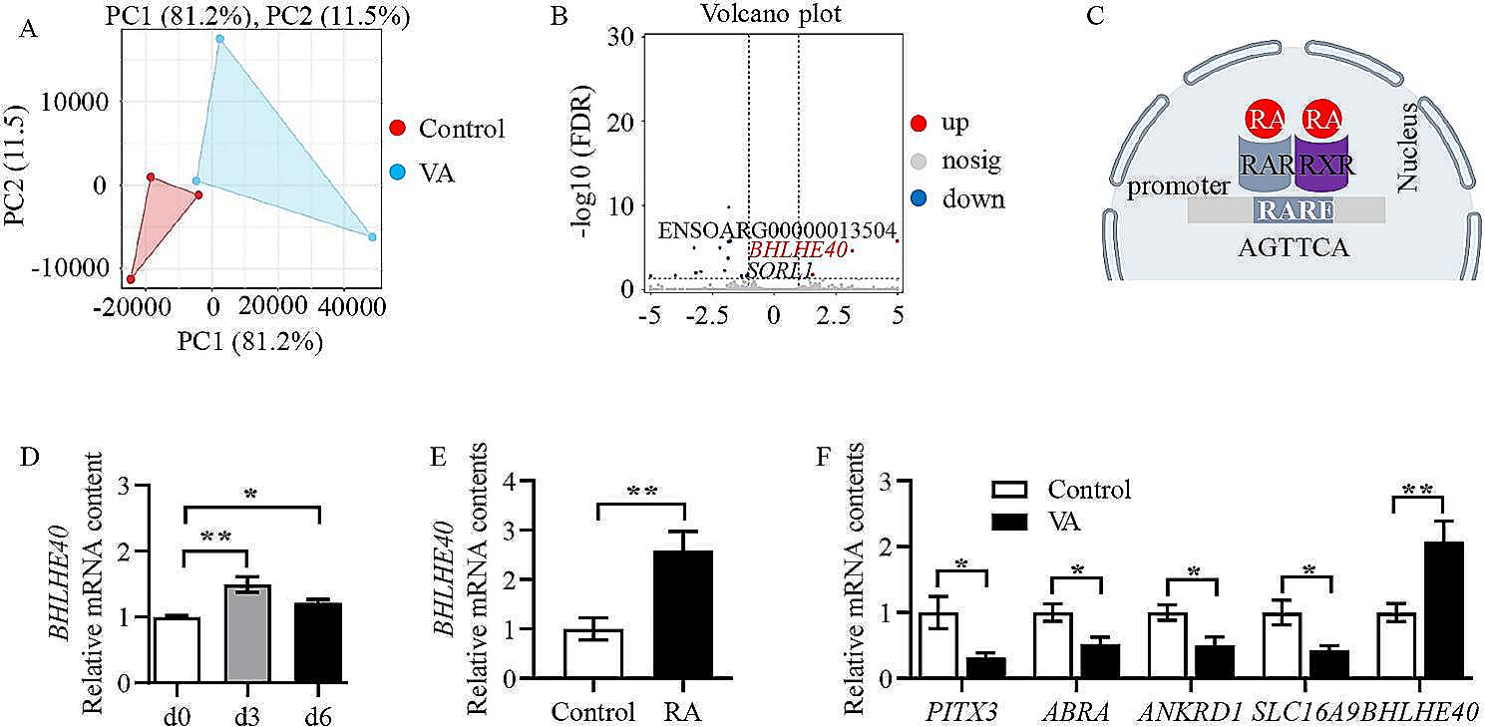
Vitamin A promotes the *BHLHE40* expression in muscle and differentiated myoblasts. (A) Principal component analysis (PCA) for all samples. (B) Differently expressed genes volcano plots for all samples. (C) Pattern map of RARE sites in the promoter region of the *BHLHE40* gene. (D) *BHLHE40* relative mRNA content at 0 d, 3 d and 6 d of sheep primary myoblasts myogenic differentiation. (E) *BHLHE40* relative mRNA content in control and RA groups of sheep primary myoblasts myogenic differentiation. (F) Relative mRNA contents of differentially expressed genes. (Mean ± SEM; *n* = 3 in each group, *P < 0.05, **P < 0.01)

Transcriptome sequencing analysis was performed to identify the target genes by which vitamin A affects skeletal muscle development. Principal component analysis (PCA) showed that a significant difference was observed between vitamin A and control groups (Fig. 1A). As shown in Fig. 1B, vitamin A was found to significantly up-regulate the expression of *BHLHE40* through RNA-seq. Further analysis revealed a fully conserved RARE site in the promoter region of the *BHLHE40* gene (Fig. 1C), which suggested that vitamin A regulated the expression of *BHLHE40*. Moreover, *BHLHE40* was found to up-regulate during myoblasts myogenic differentiation through q-PCR (Fig. 1D, *P* < 0.05), and RA increased the mRNA content of *BHLHE40 in vitro* as confirmed by q-PCR (Fig. 1E, *P* < 0.01). The q-PCR further validated the mRNA expression of *PIX3*, *ABRA*, *ANKRD1*, *SLC16A9* and *BHLHE40*. The results showed that the mRNA expression of *PIX3*, *ABRA*, *ANKRD1* and *SLC16A9* were decreased significantly (Fig. 1F, *P* < 0.05), while that of *BHLHE40* was increased significantly with VA treatment (Fig. 1F, *P* < 0.05), which were identical to the results of RNA-seq (additional file 1).

### RA inhibits the proliferation of sheep primary myoblasts through *BHLHE40*

**Fig. 2:**
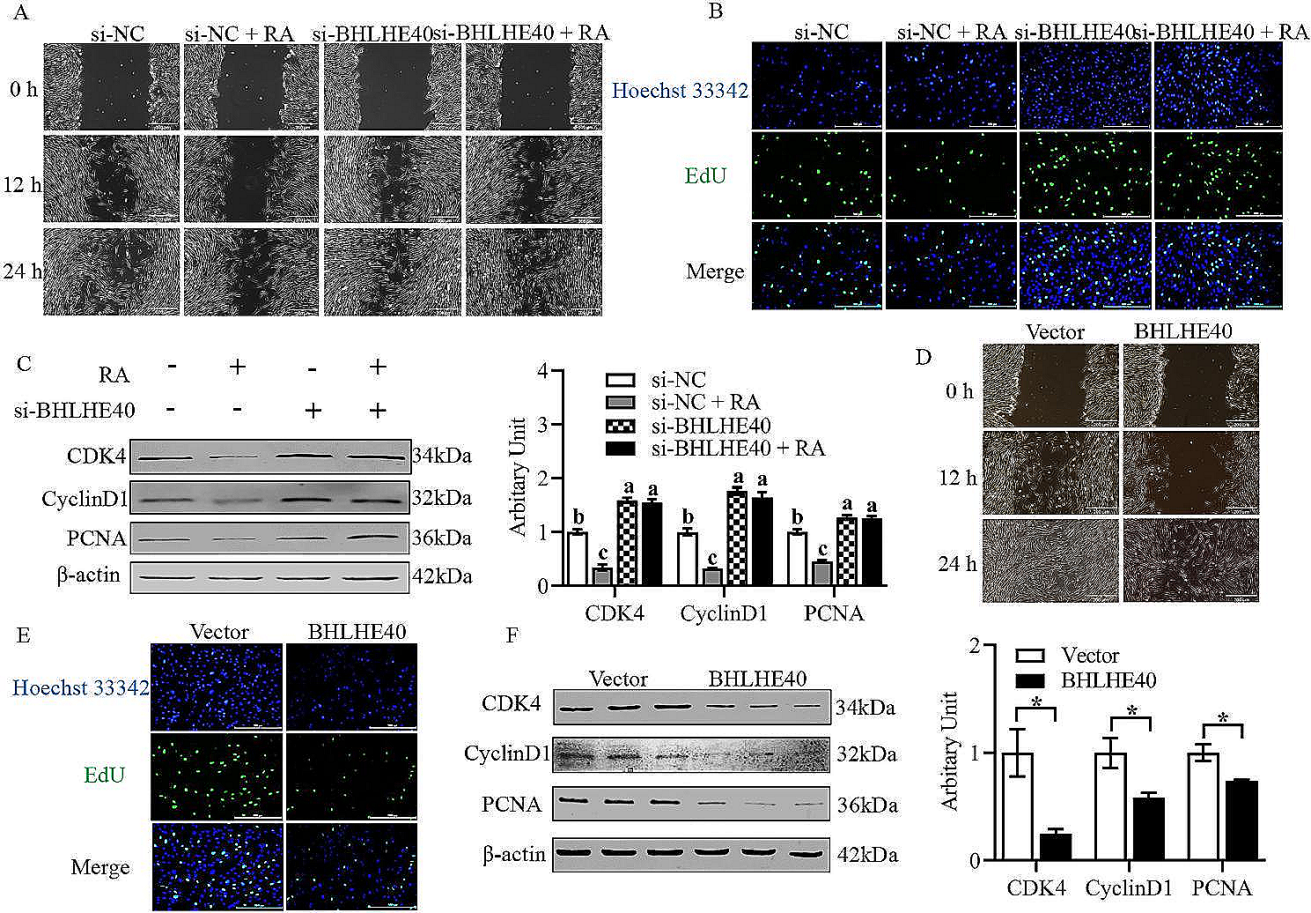
RA inhibits the proliferation of sheep primary myoblasts through *BHLHE40*. (A) Representative images of wound healing scratch assay after inhibition of *BHLHE40*. (B) Representative images of EdU staining assay after inhibition of *BHLHE40*. (C) Protein abundance of CDK4, Cyclin D1 and PCNA after inhibition of *BHLHE40*. (D) Representative images of wound healing scratch assay after overexpression of *BHLHE40*. (E) Representative images of EdU staining assay after overexpression of *BHLHE40*. (F) Protein abundance of CDK4, Cyclin D1 and PCNA after overexpression of *BHLHE40*. (Mean ± SEM; *n* = 3 in each group, *P < 0.05. Values with different letters indicated significant differences. The blots were cut prior to hybridisation with antibodies, and images of blots for all replicates were provided in the additional file 4)

As shown in Fig. 2A and B, RA was shown to significantly inhibit migration ability and EdU labeling of sheep primary myoblasts through cell scratch and EdU assays, respectively. The protein abundance of CDK4, CyclinD1 and PCNA were found to significantly decrease through western blotting (Fig. 2C, *P* < 0.05). After silencing *BHLHE40*, the migration ability and EdU labeling of sheep primary myoblasts were found to significantly enhance through cell scratch and EdU assays, respectively (Fig. 2A and B), and the protein abundance of CDK4, CyclinD1 and PCNA were found to be greatly increased through western blotting (Fig. 2C, *P* < 0.05). Silencing of *BHLHE40* with concomitant addition of RA did not restore the migration capacity, EdU labeling, and protein abundance of CDK4, CyclinD1 and PCNA in sheep primary myoblasts as revealed by cell scratch, EdU, and western blotting assays (Fig. 2A–C, *P* > 0.05). Overexpression of *BHLHE40* was found to significantly inhibit the proliferation of sheep primary myoblasts through cell scratch, EdU, and western blotting assays, including decreased cell migration ability (Fig. 2D), reduced EdU labeling (Fig. 2E), and decreased abundance of CDK4, CyclinD1 and PCNA proteins (Fig. 2F, *P* < 0.05).

### RA promotes the differentiation of sheep primary myoblasts through *BHLHE40*

**Fig. 3:**
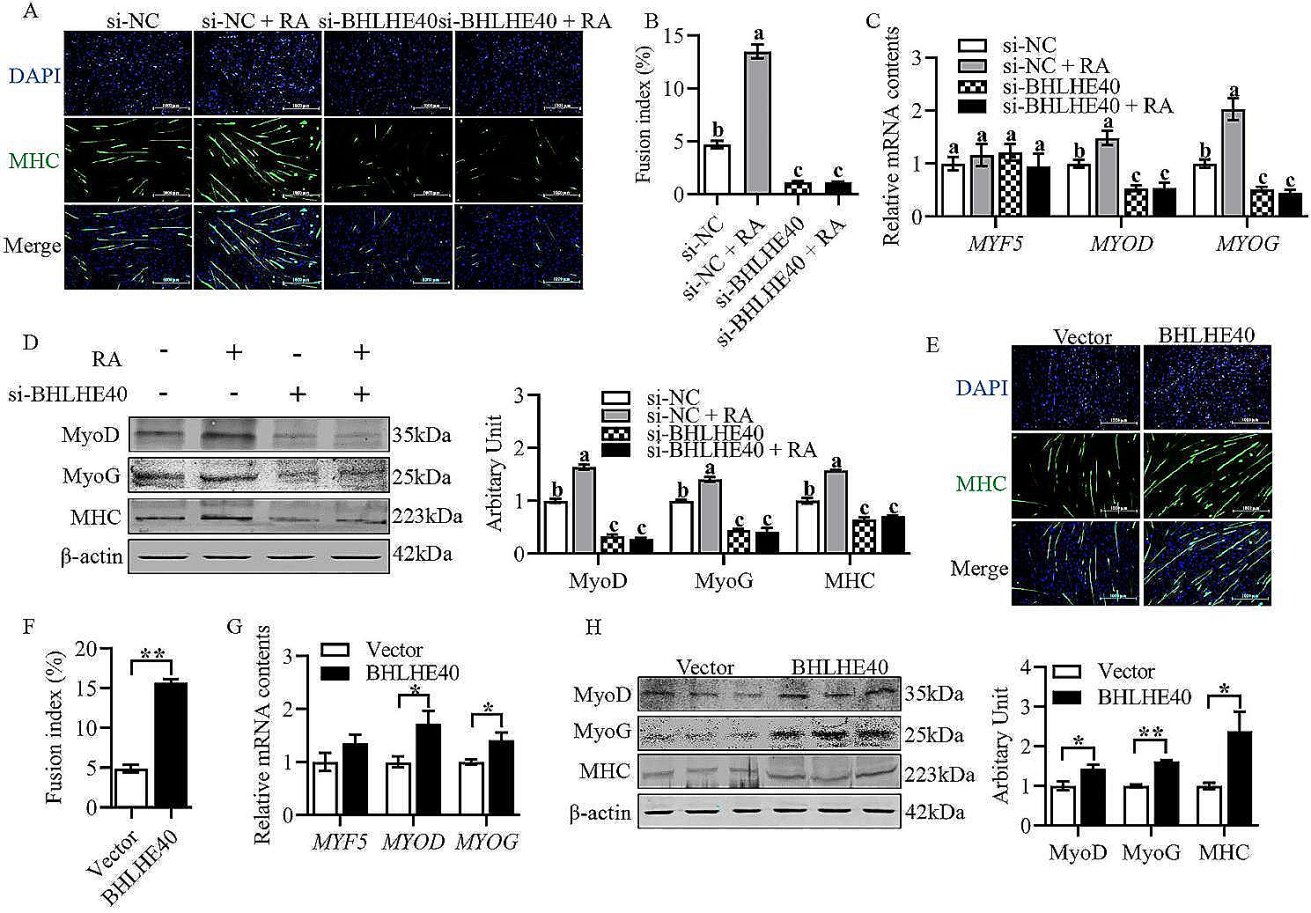
RA promotes the differentiation of sheep primary myoblasts through *BHLHE40*. (A) Representative images of MHC immunofluorescence staining after inhibition of *BHLHE40*. (B) Fusion index. (C) Relative mRNA contents of *MYF5*, *MYOD* and *MYOG* after inhibition of *BHLHE40*. (D) Protein abundance of MyoD, MyoG and MHC after inhibition of *BHLHE40*. (E) Representative images of MHC immunofluorescence staining after overexpression of *BHLHE40*. (F) Fusion index. (G) Relative mRNA contents of *MYF5*, *MYOD* and *MYOG* after overexpression of *BHLHE40*. (H) Protein abundance of MyoD, MyoG and MHC after overexpression of *BHLHE40*. (Mean ± SEM; *n* = 3 in each group, *P < 0.05, **P < 0.01. Values with different letters indicated significant differences. The blots were cut prior to hybridisation with antibodies, and images of blots for all replicates were provided in the additional file 4)

As shown in Fig. 3A and B, RA was found to significantly promote the differentiation of sheep primary myoblasts and increased the myotube fusion index through cell immunofluorescence staining assay (Fig. 3A and B, *P* < 0.05). The mRNA contents of *MYOD* and *MYOG*, and the protein abundance of MyoD, MyoG and MHC were found to increase significantly through q-PCR and western blotting (Fig. 3C and D, *P* < 0.05). After silencing *BHLHE40*, the differentiation ability of sheep primary myoblasts was found to significantly inhibit through cell immunofluorescence staining assay, and the myotube fusion index was significantly reduced (Fig. 3A and B, *P* < 0.05). The mRNA contents of *MYOD* and *MYOG*, and the protein abundance of MyoD, MyoG and MHC were found to decrease significantly through q-PCR and western blotting (Fig. 3C and D, *P* < 0.05). Silencing of *BHLHE40* with concomitant addition of RA did not restore the differentiation ability of sheep primary myoblasts, myotube fusion index, *MYOD* and *MYOG* mRNA contents and MyoD, MyoG and MHC protein abundance as revealed by cell immunofluorescence staining, q-PCR and western blotting (Fig. 3A–D, *P* > 0.05). After transfecting the cells with the *BHLHE40* overexpression plasmid, the myotube fusion index was found to significantly increase through cell immunofluorescence staining assay (Fig. 3E and F, *P* < 0.01), the relative mRNA contents of *MYOD* and *MYOG* were found to significantly increase through q-PCR (Fig. 3G, *P* < 0.05), and the protein abundance of MyoD, MyoG, and MHC were also found to significantly increase through western blotting (Fig. 3H, *P* < 0.05).

### BHLHE40 targets and inhibits *ID3* gene expression

**Fig. 4:**
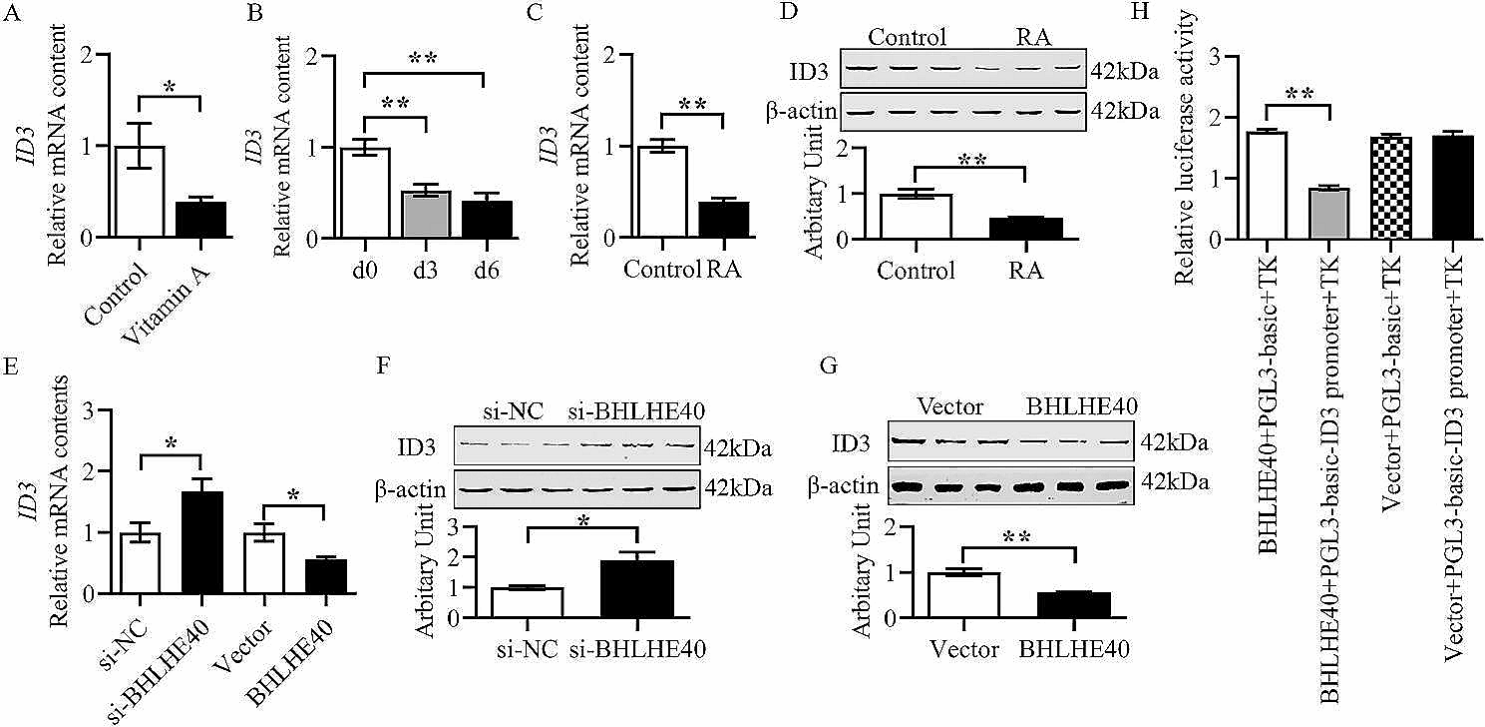
BHLHE40 targets and inhibits *ID3* gene expression. (A) Relative mRNA content of *ID3* in control and Vitamin A groups lamb. (B) Relative mRNA content of *ID3* during differentiation of myoblasts. (C) Relative mRNA content of *ID3* in control and RA groups of differentiated myoblasts. (D) Protein abundance of ID3 in control and RA groups of differentiated myoblasts. (E) Relative mRNA content of *ID3* after inhibition or overexpression of *BHLHE40*. (F) Protein abundance of ID3 after inhibition of *BHLHE40*. (G) Protein abundance of ID3 after overexpression of *BHLHE40*. (H) Dual luciferase reporter assay. (Mean ± SEM; *n* = 3 in each group, *P < 0.05, **P < 0.01. The blots were cut prior to hybridisation with antibodies, and images of blots for all replicates were provided in the additional file 4)

The intersecting genes predicted by online databases (GTRD, Cistrome Data Browser and hTFtarget) were taken as target genes of BHLHE40. Review of references revealed that *ID3* negatively regulates the differentiation process of skeletal muscle cells. In addition, the prediction by JASPAR database revealed the presence of a high-scoring binding site of BHLHE40 in the promoter region of *ID3* gene. Therefore, *ID3* was chosen as the target gene of BHLHE40 for further studies. As shown in Fig. 4A, the mRNA content of *ID3* in lambs of vitamin A group was significantly reduced as demonstrated by qPCR (Fig. 4A, *P* < 0.05). The level of *ID3* mRNA was found to be decreased with myoblast differentiation through qPCR (Fig. 4B, *P* < 0.01). Moreover, RA was shown to significantly suppress the mRNA and protein expression levels of ID3 through q-PCR and western blotting (Fig. 4C and D, *P* < 0.01). To demonstrate the targeting relationship between *BHLHE40* and *ID3*, we analyzed changes of *ID3* after silencing and overexpressing *BHLHE40*. The results revealed that ID3 mRNA and protein abundance were negatively correlated with *BHLHE40* levels through q-PCR and western blotting (Fig. 4E–G, *P* < 0.05). Furthermore, dual luciferase reporter assay showed that BHLHE40 repressed the expression of *ID3* gene (Fig. 4H, *P* < 0.01).

### *ID3* promotes the proliferation of sheep primary myoblasts

**Fig. 5:**
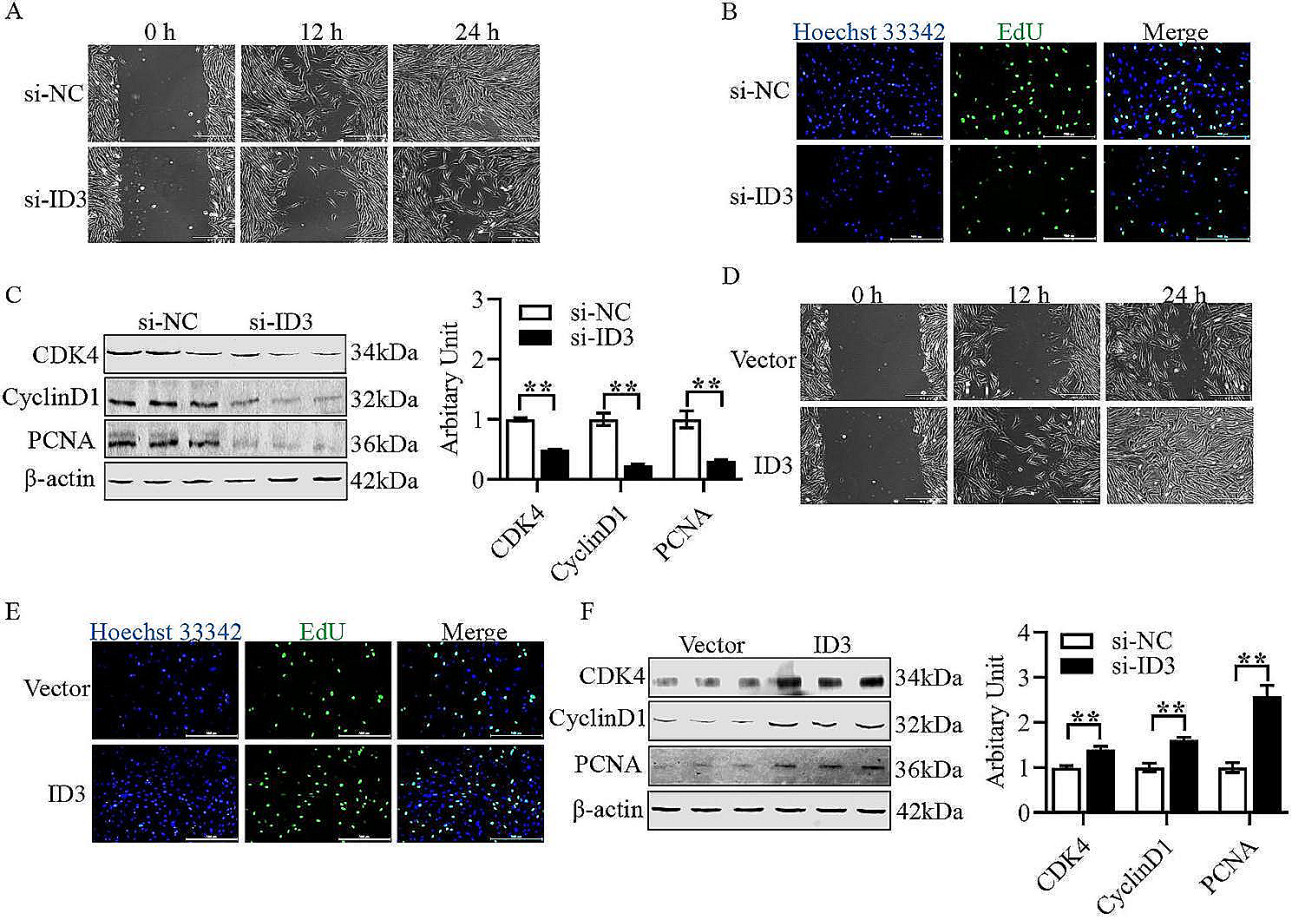
*ID3* promotes the proliferation of sheep primary myoblasts. (A) Representative images of wound healing scratch assay after inhibition of *ID3*. (B) Representative images of EdU staining assay after inhibition of *ID3*. (C) Protein abundance of CDK4, Cyclin D1 and PCNA after inhibition of *ID3*. (D) Representative images of wound healing scratch assay after overexpression of *ID3*. (E) Representative images of EdU staining assay after overexpression of *ID3*. (F) Protein abundance of CDK4, Cyclin D1 and PCNA after overexpression of *ID3*. (Mean ± SEM; *n* = 3 in each group, **P < 0.01. The blots were cut prior to hybridisation with antibodies, and images of blots for all replicates were provided in the additional file 4)

As shown in Fig. 5A and B, it was found that silencing *ID3* significantly inhibited migration ability and EdU labeling, and decreased the abundance of CDK4, CyclinD1 and PCNA of sheep primary myoblasts through cell scratch, EdU, and western blotting assays (Fig. 5C, *P* < 0.01). After transfecting *ID3* overexpression vector into the cells, the proliferation ability of myoblasts was found to be significantly elevated through cell scratch, EdU and western blotting assays, including enhanced cell migration ability (Fig. 5D), increased EdU labeling (Fig. 5E) and increased the abundance of CDK4, CyclinD1 and PCNA of sheep primary myoblasts (Fig. 5F, *P* < 0.01).

### *ID3* inhibits the differentiation of sheep primary myoblasts

**Fig. 6:**
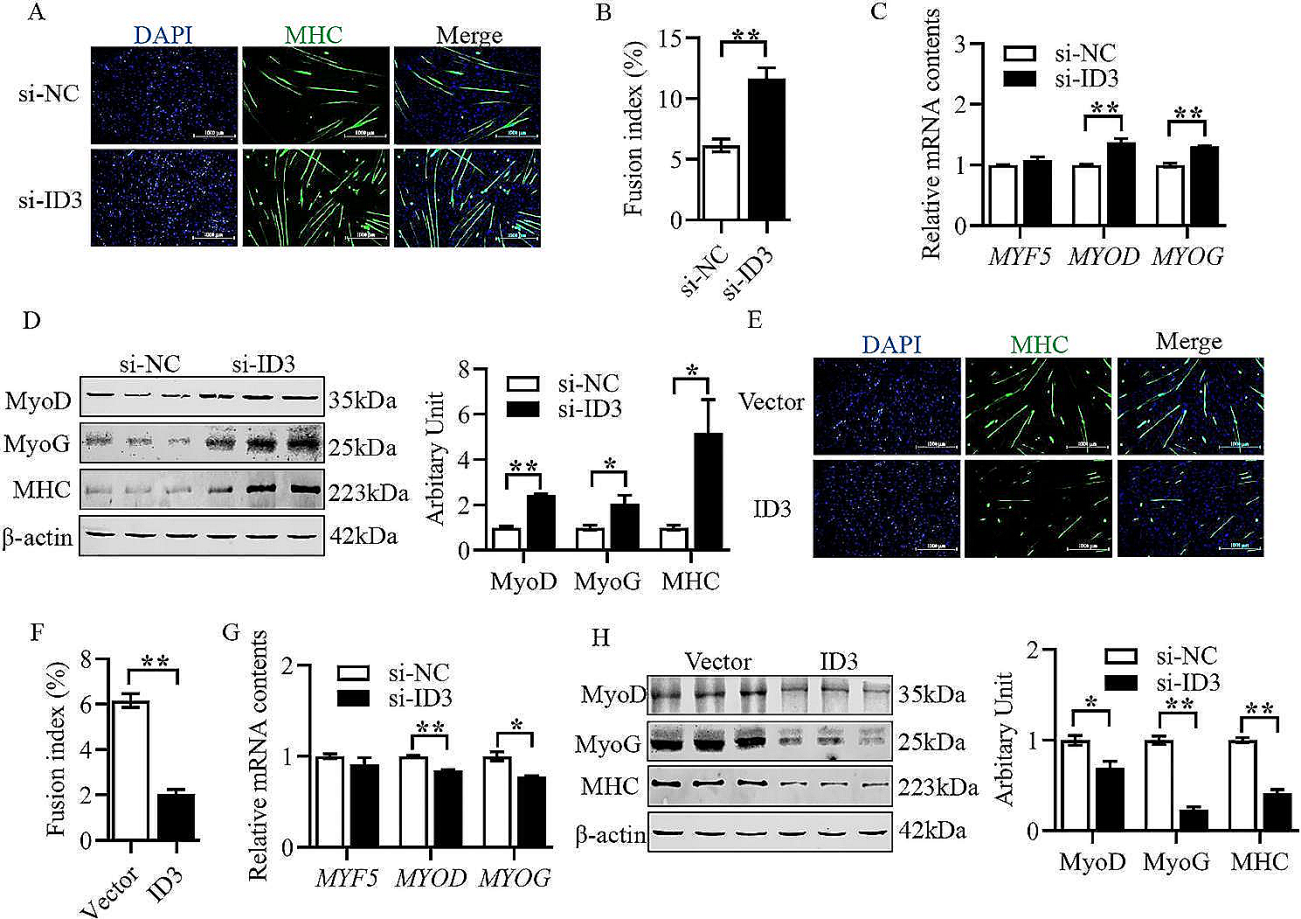
*ID3* inhibits the differentiation of sheep primary myoblasts. (A) Representative images of MHC immunofluorescence staining after inhibition of *ID3*. (B) Fusion index. (C) Relative mRNA contents of *MYF5*, *MYOD* and *MYOG* after inhibition of *ID3*. (D) Protein abundance of MyoD, MyoG and MHC after inhibition of *ID3*. (E) Representative images of MHC immunofluorescence staining after overexpression of *ID3*. (F) Fusion index. (G) Relative mRNA contents of *MYF5*, *MYOD* and *MYOG* after overexpression of *ID3*. (H) Protein abundance of MyoD, MyoG and MHC after overexpression of *ID3.* (Mean ± SEM; *n* = 3 in each group, *P < 0.05, **P < 0.01. The blots were cut prior to hybridisation with antibodies, and images of blots for all replicates were provided in the additional file 4)

As shown in Fig. 6A and B, the sheep primary myoblasts differentiation ability and myotube fusion index was found to significantly increase after silencing *ID3* through cell immunofluorescence staining assay (Fig. 6A and B, *P* < 0.01). It was that the mRNA contents of *MYOD* and *MYOG* were significantly elevated through q-PCR (Fig. 6C, *P* < 0.01), as was the protein abundance of MyoD, MyoG and MHC through western blotting (Fig. 6D, *P* < 0.05). In addition, after transfecting *ID3* overexpression vector into the cells, the differentiation ability and myotube fusion index of sheep primary myoblasts were found to be significantly reduced through cell immunofluorescence staining assay (Fig. 6E and F, *P* < 0.01), the mRNA contents of *MYOD* and *MYOG* were found to be significantly decreased through q-PCR (Fig. 6G, *P* < 0.05), and the protein abundance of MyoD, MyoG, and MHC were also found to be significantly decreased through western blotting (Fig. 6H, *P* < 0.05).

## Discussion

Sheep as a meat animal is widely favored by the public for its rich nutritional value [28–31]. In recent years, lamb production has increased in many countries and regions to meet consumer demand [32, 33]. Therefore, understanding the mechanisms of skeletal muscle development is urgently needed to improve meat production in sheep. In previous studies, we found that intramuscular injection of vitamin A promotes muscle development in lambs [17]. However, molecular regulation during skeletal muscle development is highly sophisticated and complex. For example, the sonic hedgehog (Shh), notch and wnt signaling are able to determine the direction of myogenic progenitor cell differentiation [34–36]. Myogenic regular factors (MRFs) are essential in myogenesis, and when myoblasts exit the cell cycle, they fuse to form multinucleated myotubes under the regulation of MRFs, and further fuse to form mature myofibers. Moreover, interactions among numerous transcription factors are involved in regulating skeletal muscle formation and development [37, 38].

In the current study, to investigate the mechanisms that vitamin A affects skeletal muscle development, transcriptome sequencing technology was used to sequence skeletal muscle tissues from 3-week-old lambs. Among the differentially expressed genes, vitamin A was found to upregulate BHLHE40 expression through analysis and screening, and this gene is believed to be involved in the control of cell differentiation. It has been shown that stra13 (BHLHE40) promotes myoblast differentiation by antagonizing Notch signaling [39]. Moreover, there are at least 2 RA-responsive regions in the nucleotide sequence of the *SHARP-2* (*BHLHE40*) gene, which means that RA is able to stimulate the transcription of the *SHARP-2* gene [40]. Consistently, the mRNA level of *BHLHE40* was significantly increased after sheep primary myoblasts differentiation. Further analysis revealed the presence of a binding site for RARE in the promoter region of the *BHLHE40* gene. In addition, RA greatly increased the mRNA content of *BHLHE40* during sheep primary myoblasts myogenic differentiation in this study, implying that vitamin A promoted muscle development by up-regulating *BHLHE40* expression.

RA, a metabolite of vitamin A, is an important factor involved in cell growth and differentiation [41]. Currently, there is increasing evidence that RA inhibits cell proliferation and promotes cell differentiation in different tissues or cells [27, 42–44]. More importantly, BHLHE40, a transcriptional repressor, has been shown to be a target gene of RA in P19 cells [45], which is consistent with the sequencing results of this study. In addition, *Stra13* was shown to inhibit myoblast proliferation and promote myoblast differentiation in mice [39]. In the present experiments, silencing of *BHLHE40* promoted myoblast proliferation, and silencing of BHLHE40 with concomitant addition of RA did not attenuate the proliferative capacity of myoblasts. In contrast, overexpression of *BHLHE40* significantly inhibited myoblast proliferation. Thus, the above results suggested that the inhibition of RA on myoblast proliferation was realized through *BHLHE40*. Normally, during cell differentiation, the proliferative capacity is progressively limited and eventually exits the cell cycle [46]. For skeletal myogenesis, where proliferation and differentiation of myoblasts are opposite processes, overexpression of cyclin D1 inhibits MyoD activity [47]. Consistently, silencing of *BHLHE40* inhibited myoblast differentiation, and silencing of *BHLHE40* with concomitant addition of RA did not attenuate the inhibitory effect on myoblast differentiation. However, overexpression of *BHLHE40* promoted myoblast differentiation in this research. Hence, the above findings suggested that RA promoted myoblast differentiation by upregulating *BHLHE40*.

Transcription factors are a class of proteins that regulate gene expression [48]. BHLHE40 belongs to the class B helix-loop-helix proteins, which contain an orange (O) domain, and is a typical transcriptional repressor [49]. BHLHE40 not only binds to the E-box sequence of genes to inhibit the transcription of target genes, but also inhibits the transcription of target genes by interacting with other transcription factors [50]. In this study, the target genes of BHLHE40 were predicted by online databases (GTRD, Cistrome Data Browser and hTFtarget databases). Subsequently, prediction and analysis using the JASPAR database revealed the presence of a high-score binding site for BHLHE40 in the promoter region of *ID3* gene. Numerous researches have shown that *ID3* gene is involved in a variety of cellular processes, including cell proliferation, differentiation, apoptosis and tumor transformation [51–53]. The present experiment revealed that the mRNA level of *ID3* was significantly decreased in the skeletal muscle of vitamin A group lambs, and the mRNA level of *ID3* was also significantly decreased with the differentiation of sheep primary myoblasts. Moreover, the mRNA and protein abundance of ID3 was inversely correlated with *BHLHE40* by silencing and overexpressing *BHLHE40*, suggesting that BHLHE40 may negatively regulate *ID3* expression. Actually, many researchers have shown that BHLHE40 functions as a nuclear transcriptional repressor. For example, St-Pierre et al., showed that Stra13 directly represses the transcription of the class B E-box and related sites [54]. It has also been reported that DEC1 represses the expression of DEC2 through binding to the proximal promoter E-box [55]. Since the alterations in myoblast proliferation and differentiation were caused by changes in the *ID3* gene, we analyzed the effect of RA on *ID3* gene levels, and the results corresponded to the in vivo experiments. This is consistent with previous findings where López-Carballo et al., found a significant reduction in *ID3* mRNA levels during RA-induced differentiation of SH-SY5Y neuroblastoma cells [56]. Subsequently, a dual luciferase reporter assay confirmed that BHLHE40 inhibits the activity of the *ID3* gene promoter and represses the transcription of *ID3* gene. During myogenesis, myogenic regulators are highly coordinated interplay with the class I E proteins and class V Id proteins in the differentiation of myoblasts [57]. For ID family members (ID1, ID2, ID3 and ID4), *ID3* has the highest expression in myoblasts [58], implying that *ID3* is very important for myoblast proliferation and differentiation. In C2C12 cells, *ID3* expression delays cell exit from the cell cycle, causing inhibition of cell differentiation [11, 59]. Consistently, our experiments showed that silencing *ID3* inhibited myoblast proliferation and promoted myoblast differentiation. Accordingly, overexpression of *ID3* showed the opposite result. However, there were many target genes of BHLHE40 predicted from online databases. Further experiments are needed to demonstrate whether BHLHE40 may be involved in regulating more genes in the process of vitamin A promoted sheep primary myoblasts myogenic differentiation.

The current results demonstrated that RA inhibited myoblast proliferation and promoted myoblast differentiation through BHLHE40-targeted inhibition of *ID3* expression, which provided a scientific basis for understanding skeletal muscle development in sheep. Moreover, this research could provide a reference for the fields of muscle tissue regeneration and repair, as well as the treatment of muscle diseases and injuries.

## Conclusion

Intramuscular injection of vitamin A in newborn lambs inhibited myoblasts proliferation and promoted myoblasts myogenic differentiation by inhibiting *ID3* gene transcription through up-regulation of BHLHE40 expression, ultimately affected skeletal muscle development of lambs.

### Electronic supplementary material

Below is the link to the electronic supplementary material.

Supplementary Material 1

Supplementary Material 2

Supplementary Material 3

Supplementary Material 4

## Data Availability

The datasets presented in this study can be found in online repositories (https://ngdc.cncb.ac.cn/gsa/). The name of the repositories and accession number can be found below: Genome Sequence Archive (GSA), accession number PRJCA021156 (*n*=6).

## Abbreviations

RA: Retinoic acid
RNA-Seq: Transcriptome sequencing
LD: Longissimus dorsi
ID3: DNA binding inhibitor 3
PCR: Polymerase chain reaction
PCA: Principal component analysis
DEG: Differentially expressed gene
FDR: False discovery rate
DMEM: Dulbecco’s Modified Eagle’s medium
FBS: Fetal bovine serum
EdU: 5-ethynyl-2’-deoxyuridine
