# Supplementary material for: Vitamin a potentiates sheep myoblasts myogenic differentiation through BHLHE40-modulated ID3 expression
Source: BMC Genomics. 2024 Mar 5;25:244. doi: 10.1186/s12864-024-10161-0 (PMC10913236; doi:10.1186/s12864-024-10161-0)
Supplement: Supplementary file 2 — Supplementary Material 2 [file 12864_2024_10161_MOESM2_ESM.docx]

**[BMC Genomics](https://bmcgenomics.biomedcentral.com/)**

**Vitamin A promotes sheep primary myoblasts myogenic differentiation through BHLHE40-modulated *DNA binding inhibitor 3* expression**

**Supplementary materials 2:**

Sheep BHLHE40 gene overexpression plasmid construction

1 Vector information

PGMLV-CMV-MCS-3×Flag-EF1-ZsGreen1-T2A-Puro


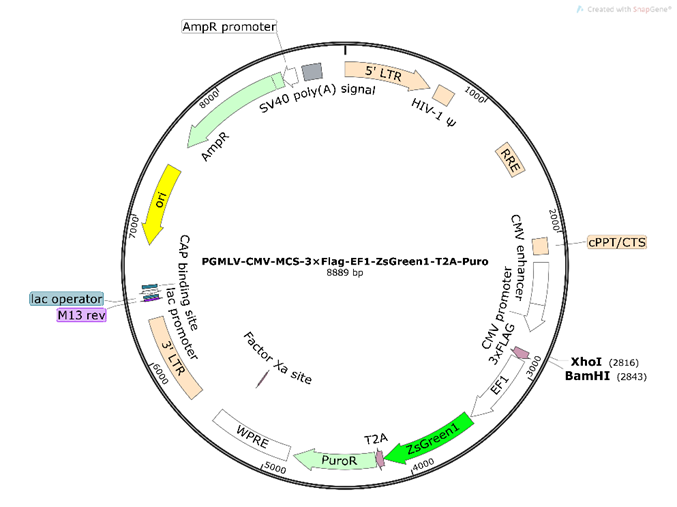


2 Primer informaton

| 71180FW-83287: GAATTCGAAGTATACCTCGAGGCCA |
| --- |
| 71180RW-83288: ATGGTCTTTGTAGTCGGATCCGTCT |

3 Restriction endonucleases information

XhoI

BamHI

4 The target fragment was amplified and ligated to the double digested vector by seamless cloning. It was then transformed into competent cells for culture, and finally the clones were selected for sequencing and identification.

5 Results

Plasmid Name

| PGMLV-CMV-Sheep_BHLHE40-3×Flag-EF1-ZsGreen1-T2A-Puro |
| --- |
| 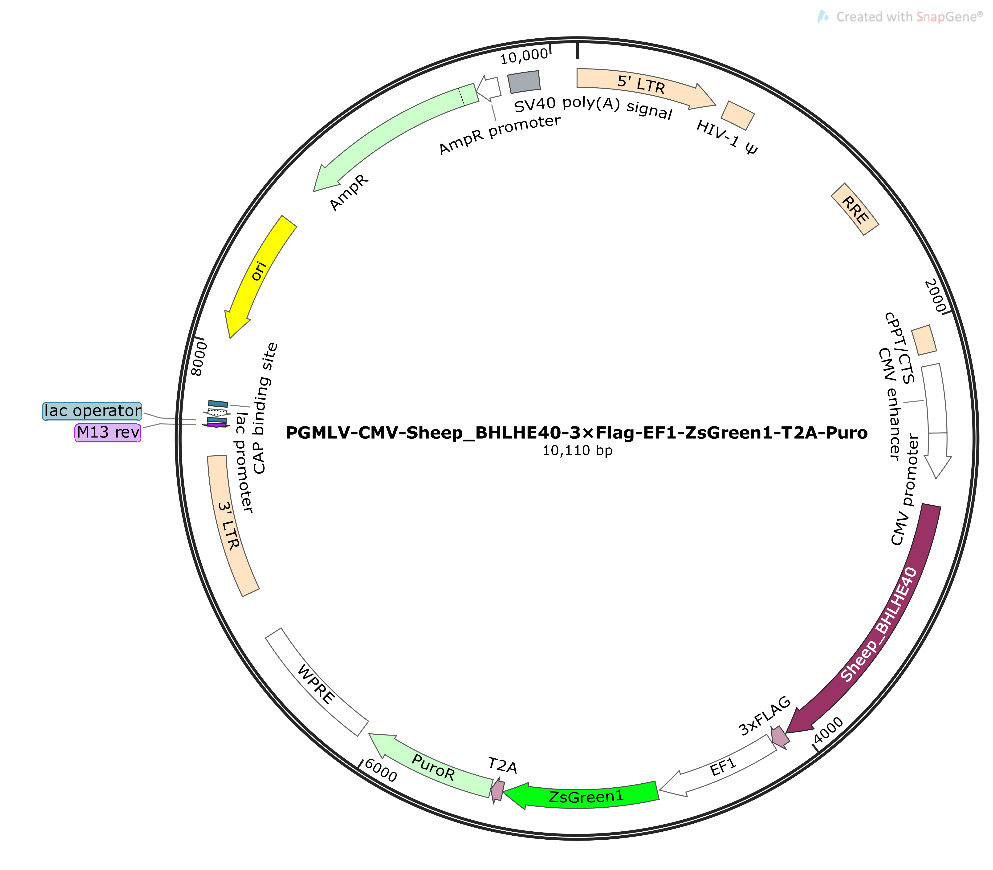 |

After comparison, the sequence of the inserted fragment in the recombinant clone was identical with the sequence of the target fragment, so the vector construction was successful.

Sheep ID3 gene overexpression plasmid construction

1 Vector information

PGMLV-CMV-MCS-3×Flag-EF1-ZsGreen1-T2A-Puro


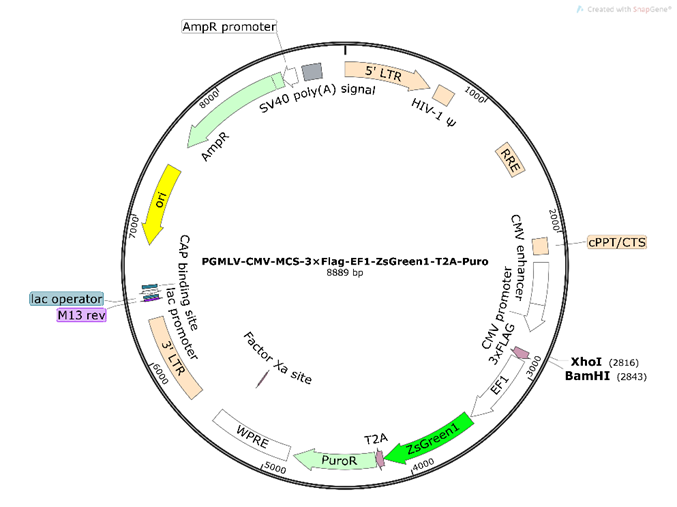


| 2 Primer information |
| --- |
| 78779FW-90255: GAATTCGAAGTATACCTCGAGGCCA |
| 78779RW-90256: ATGGTCTTTGTAGTCGGATCCGTG |

3 Restriction endonucleases information

XhoI

BamHI

4 The target fragment was amplified and ligated to the double digested vector by seamless cloning. It was then transformed into competent cells for culture, and finally the clones were selected for sequencing and identification.

5 Results

Plasmid Name

PGMLV-CMV-Sheep_ID3-3×Flag-EF1-ZsGreen1-T2A-Puro

| 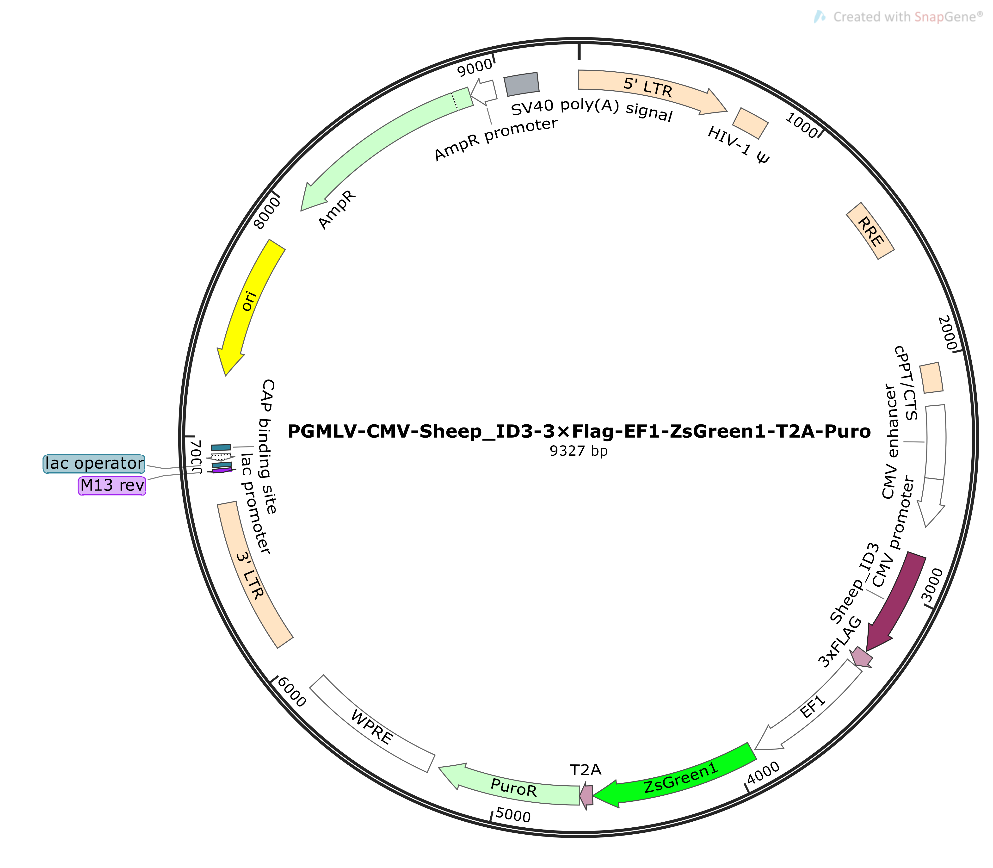 |
| --- |

After comparison, the sequence of the inserted fragment in the recombinant clone was identical with the sequence of the target fragment, so the vector construction was successful.
