# Supplementary material for: Vitamin a potentiates sheep myoblasts myogenic differentiation through BHLHE40-modulated ID3 expression
Source: BMC Genomics. 2024 Mar 5;25:244. doi: 10.1186/s12864-024-10161-0 (PMC10913236; doi:10.1186/s12864-024-10161-0)

1. Protein Marker (#, MF212 (10-180 kDa) and MF290-plus (10-310 kDa); M5 Prestained Protein Ladder (Mei5 Biotechnology Co.,Ltd, Beijing, China) we used were shown below:


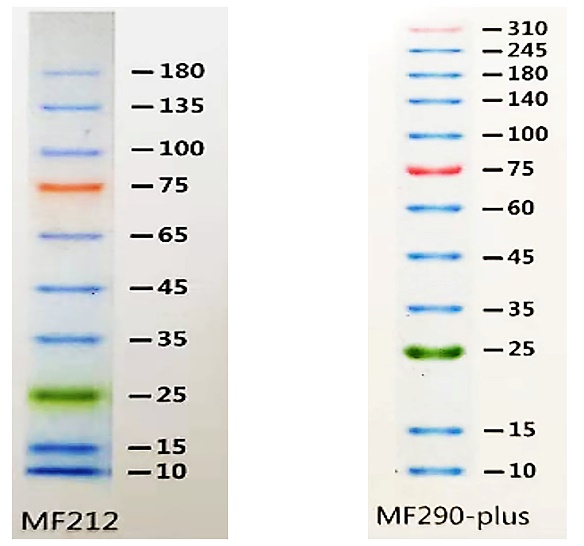


2. The original gels of the western blots in manuscript. (M: protein marker, L: protein lanes)

Fig.2C L1: si-NC, L2: si-NC+RA, L3: si-BHLHE40, L4: si-BHLHE40+RA.

CDK4


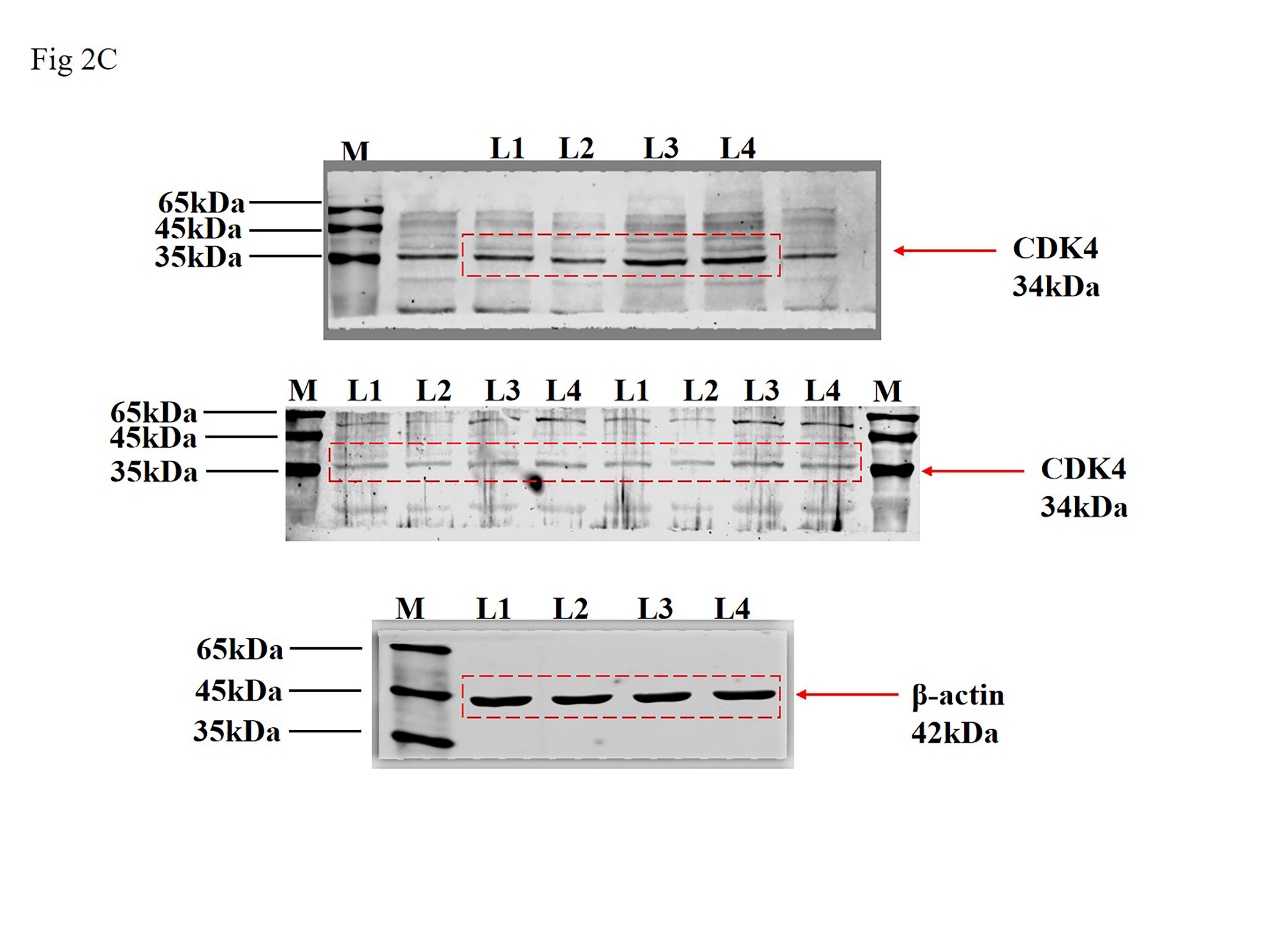


CyclinD1


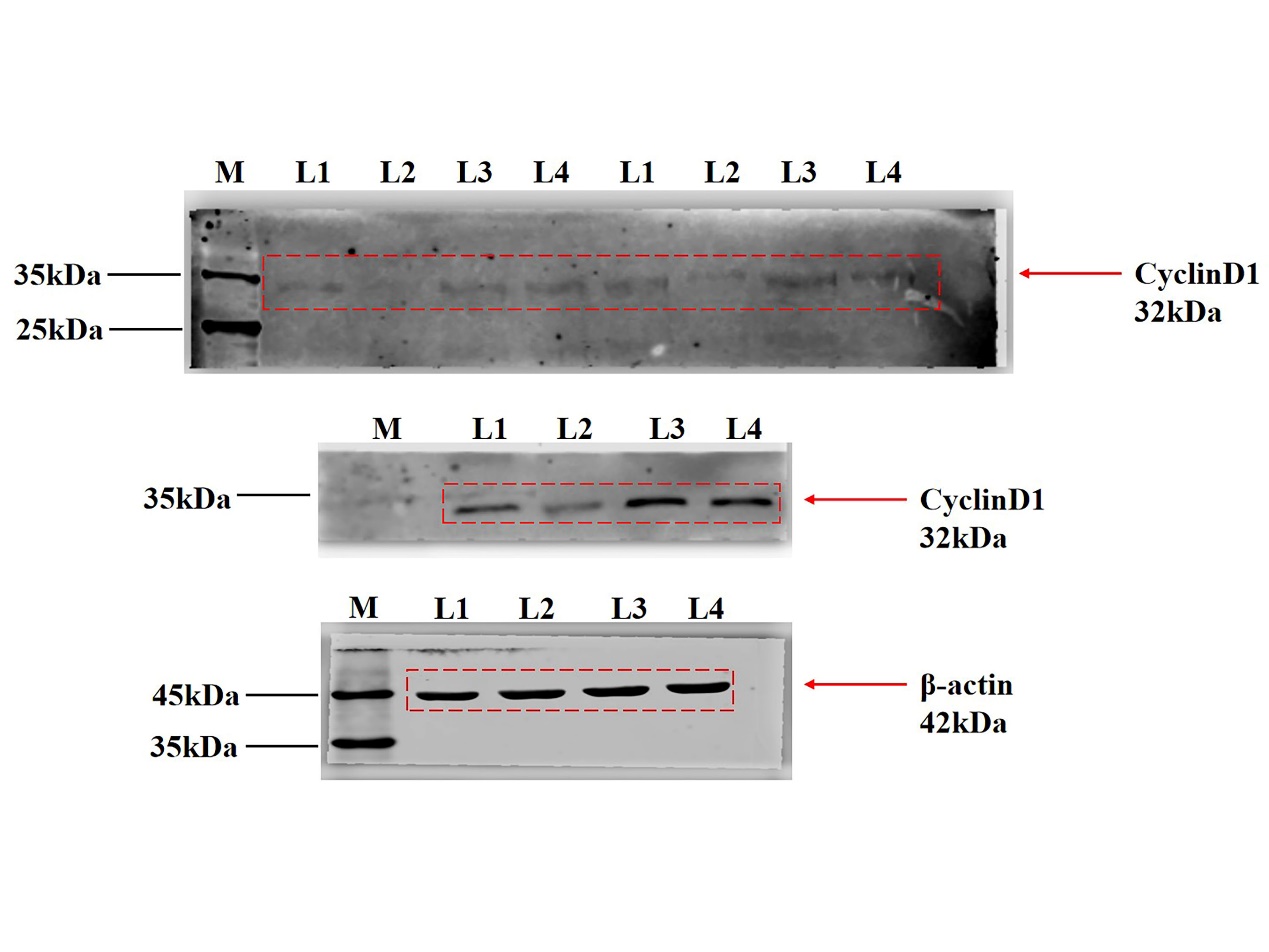


PCNA


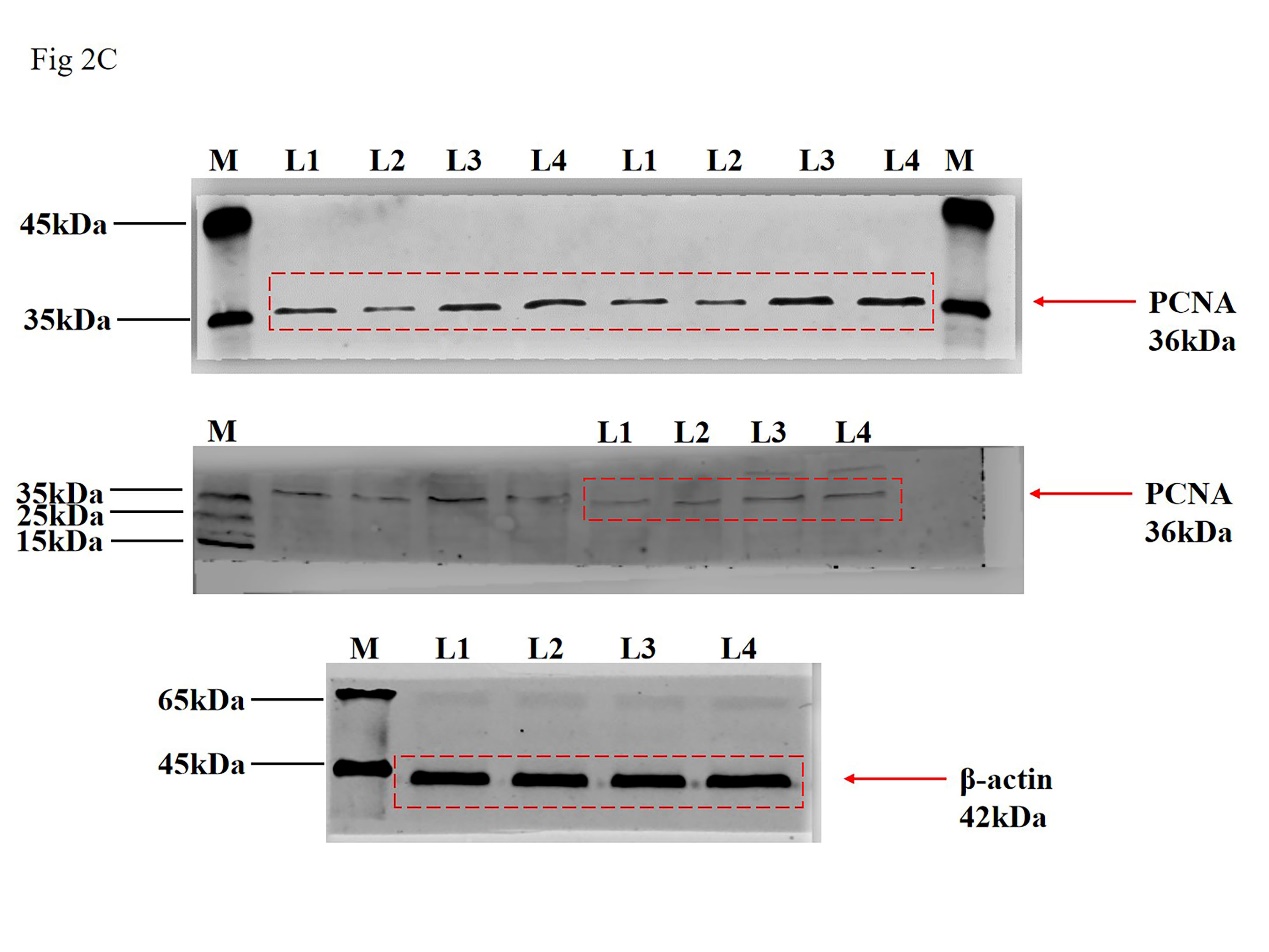


Fig.2F L1-L3: Vector; L4-L6: BHLHE40.

CDK4


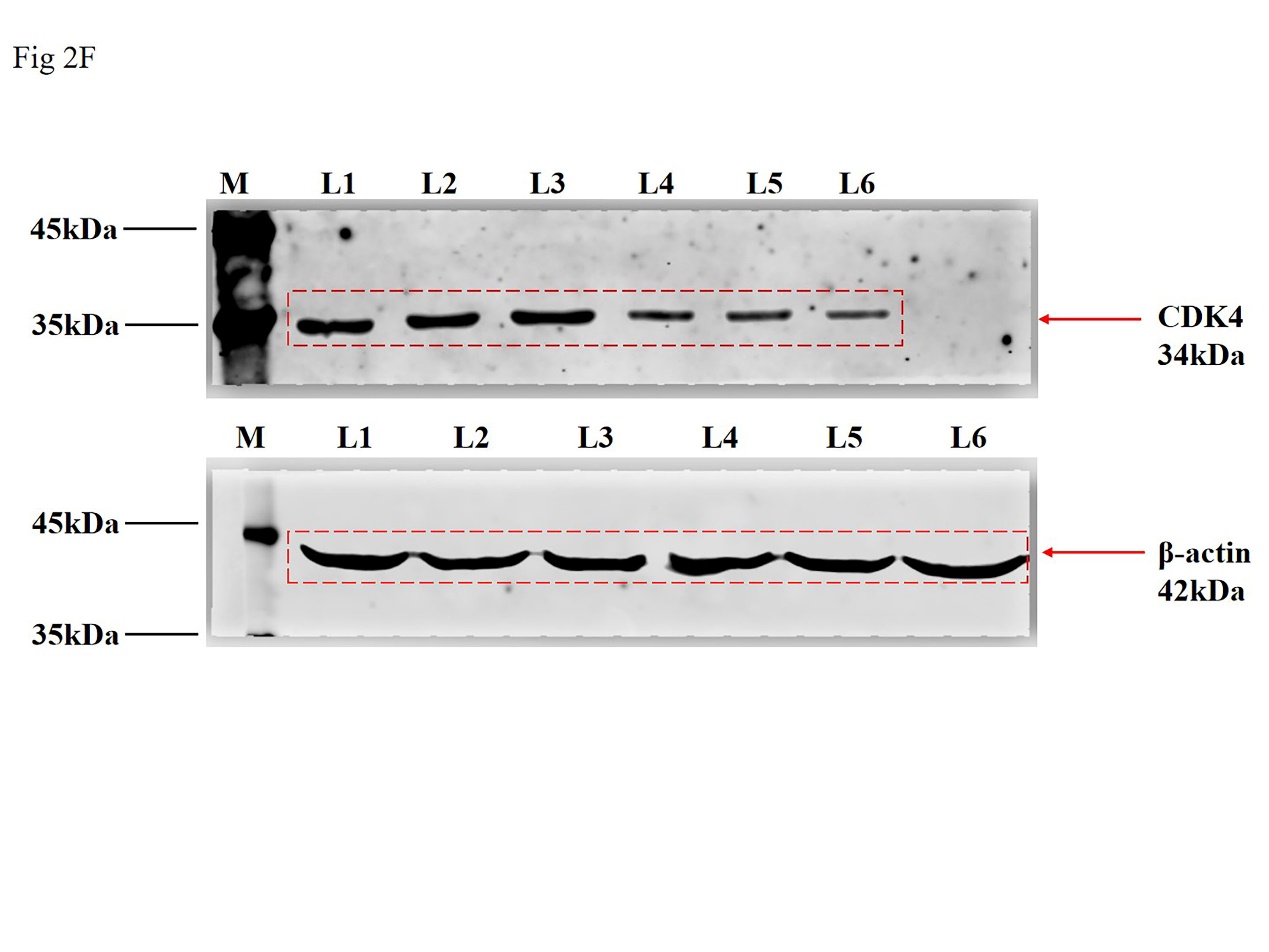


CyclinD1


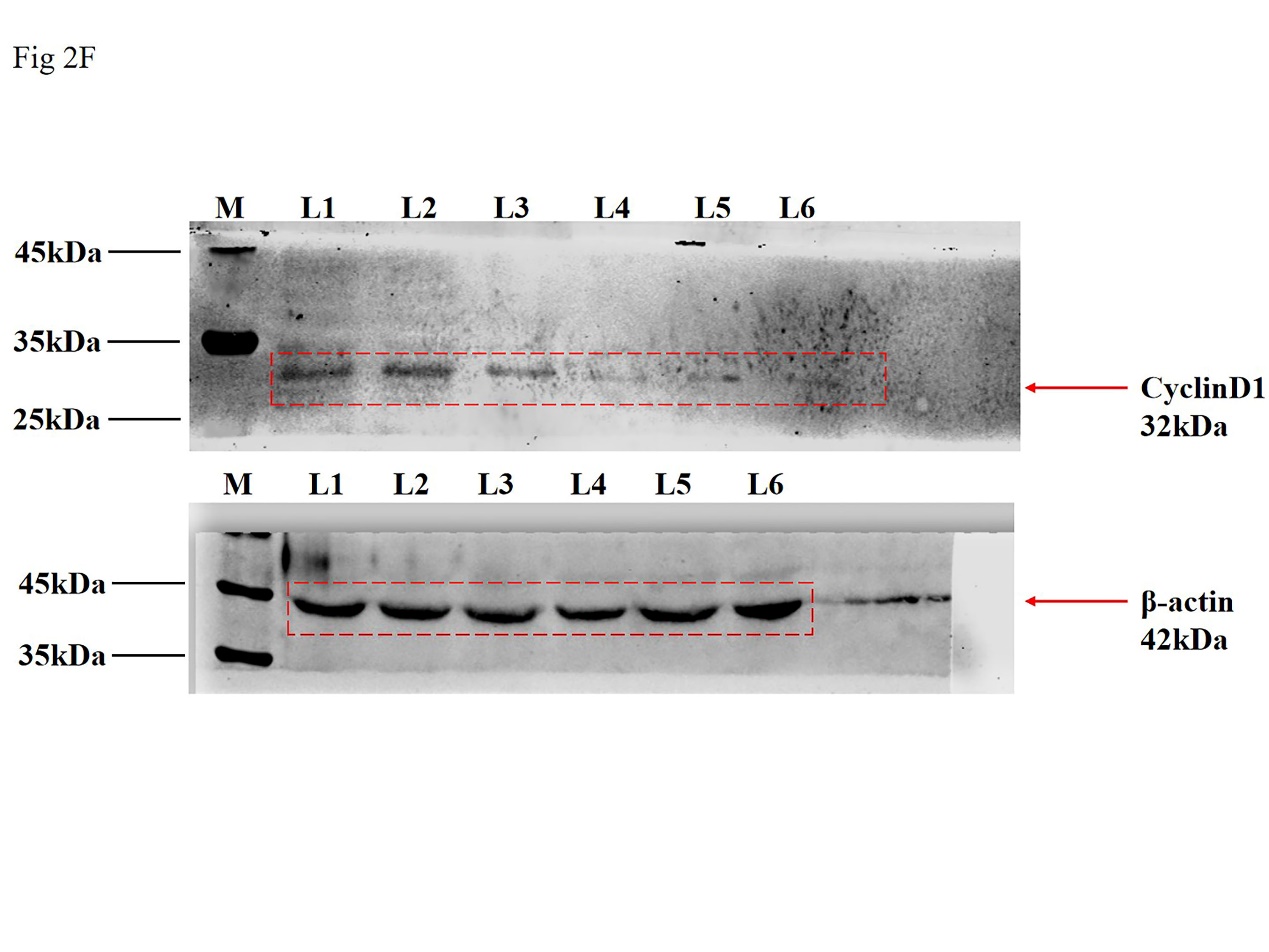


PCNA


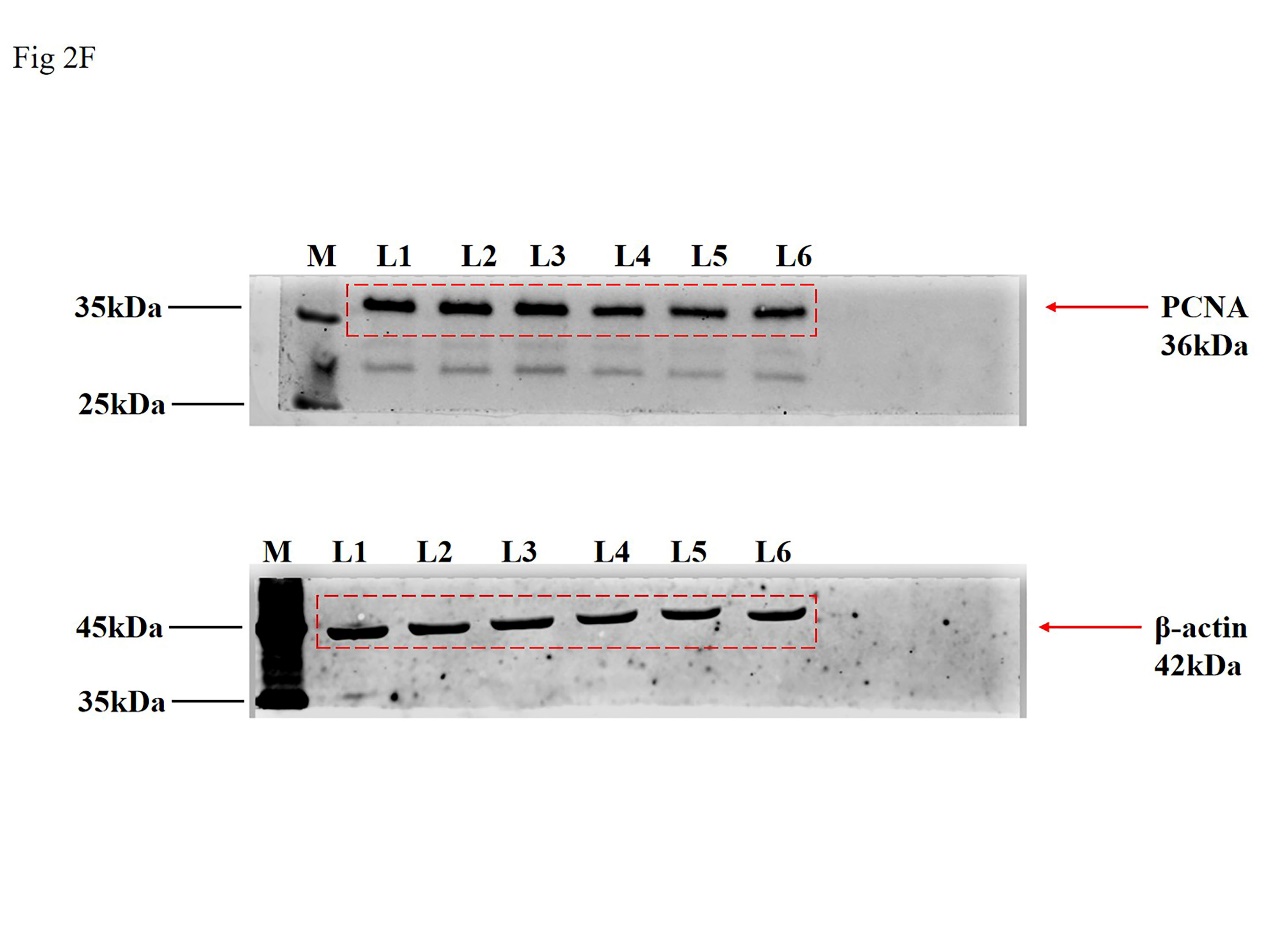


Fig.3D L1: si-NC, L2: si-NC+RA, L3: si-BHLHE40, L4: si-BHLHE40+RA.

MyoD


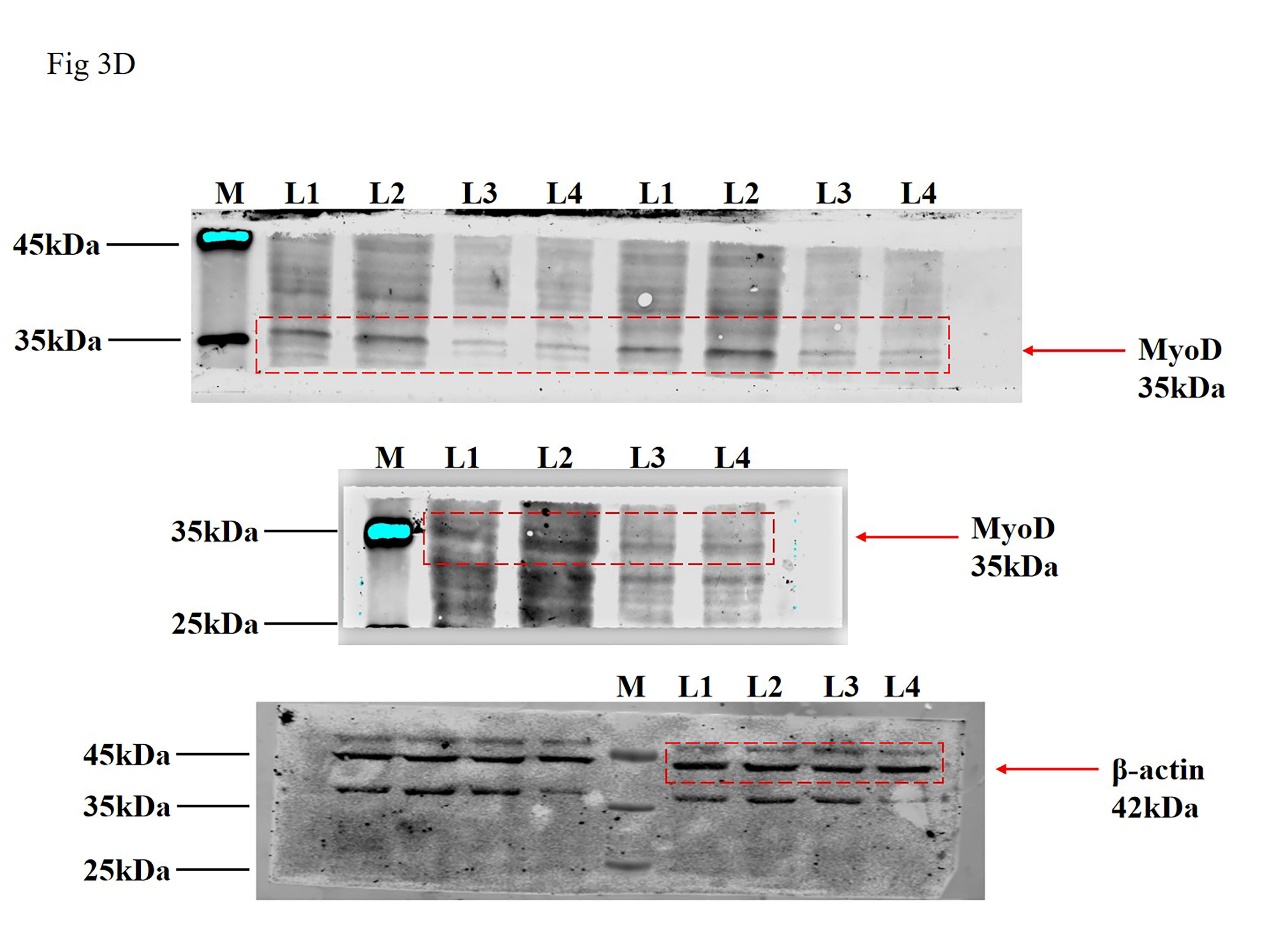


MyoG


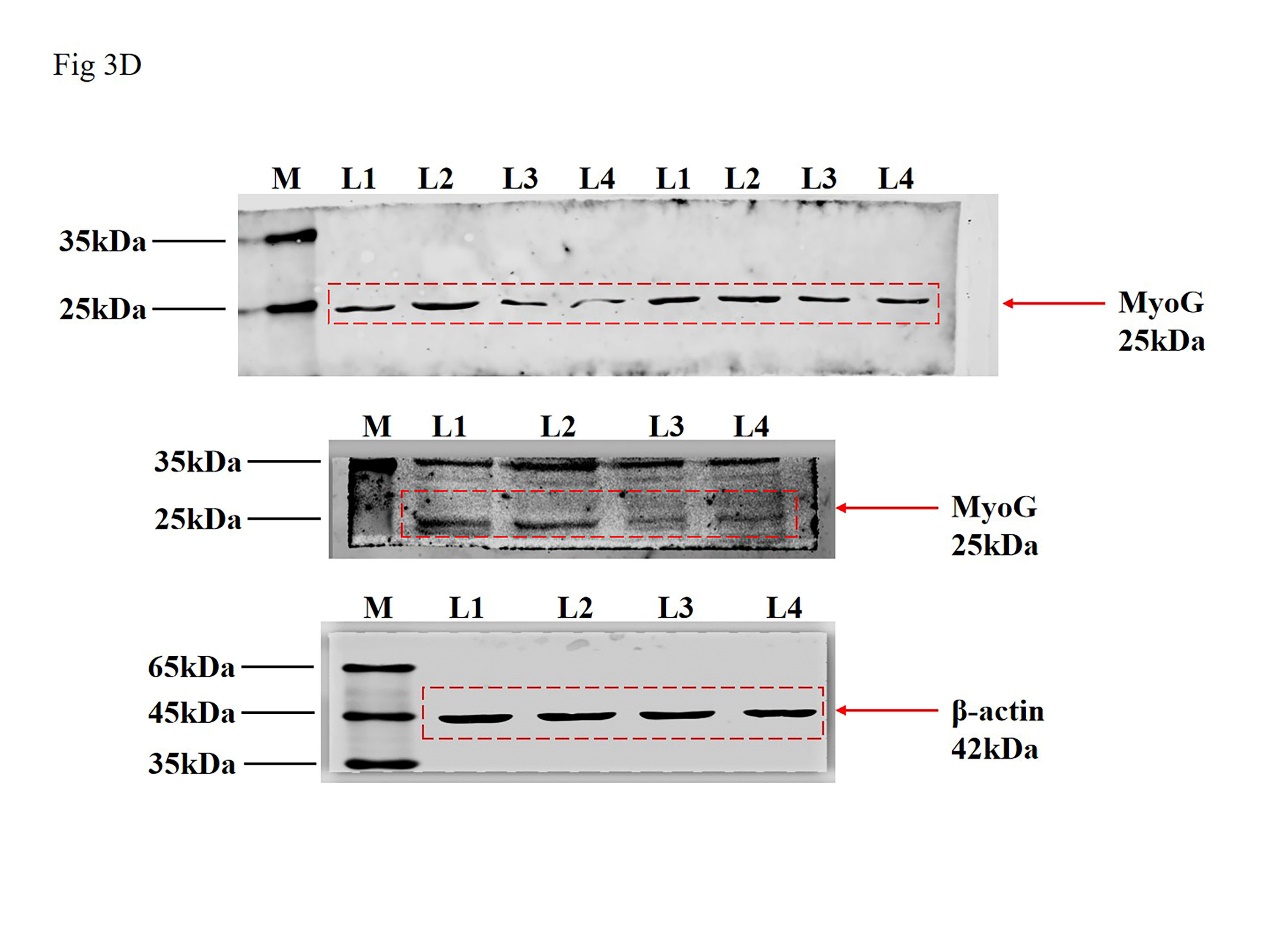


MHC


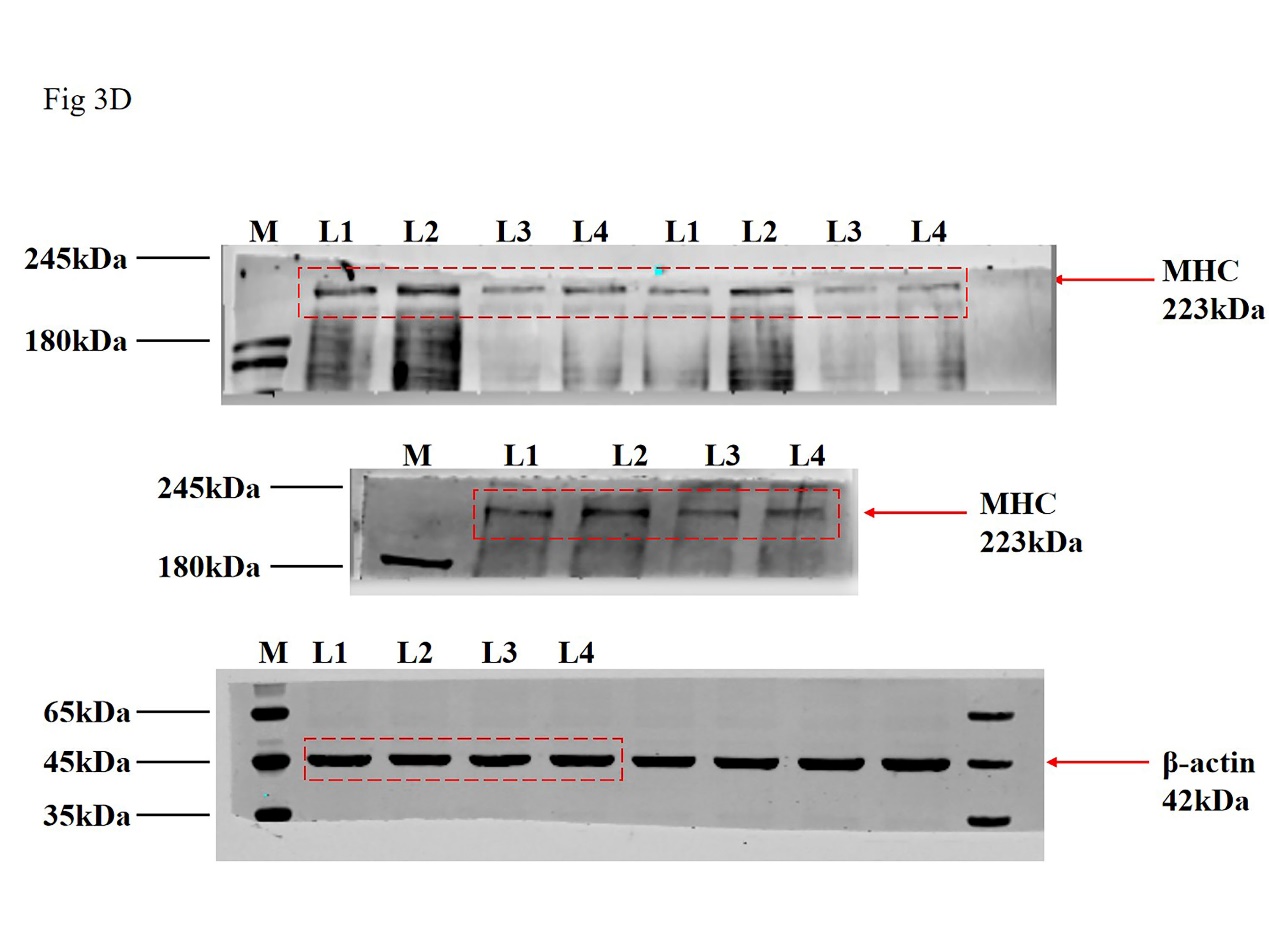


Fig.3H L1-L3: Vector; L4-L6: BHLHE40.

MyoD


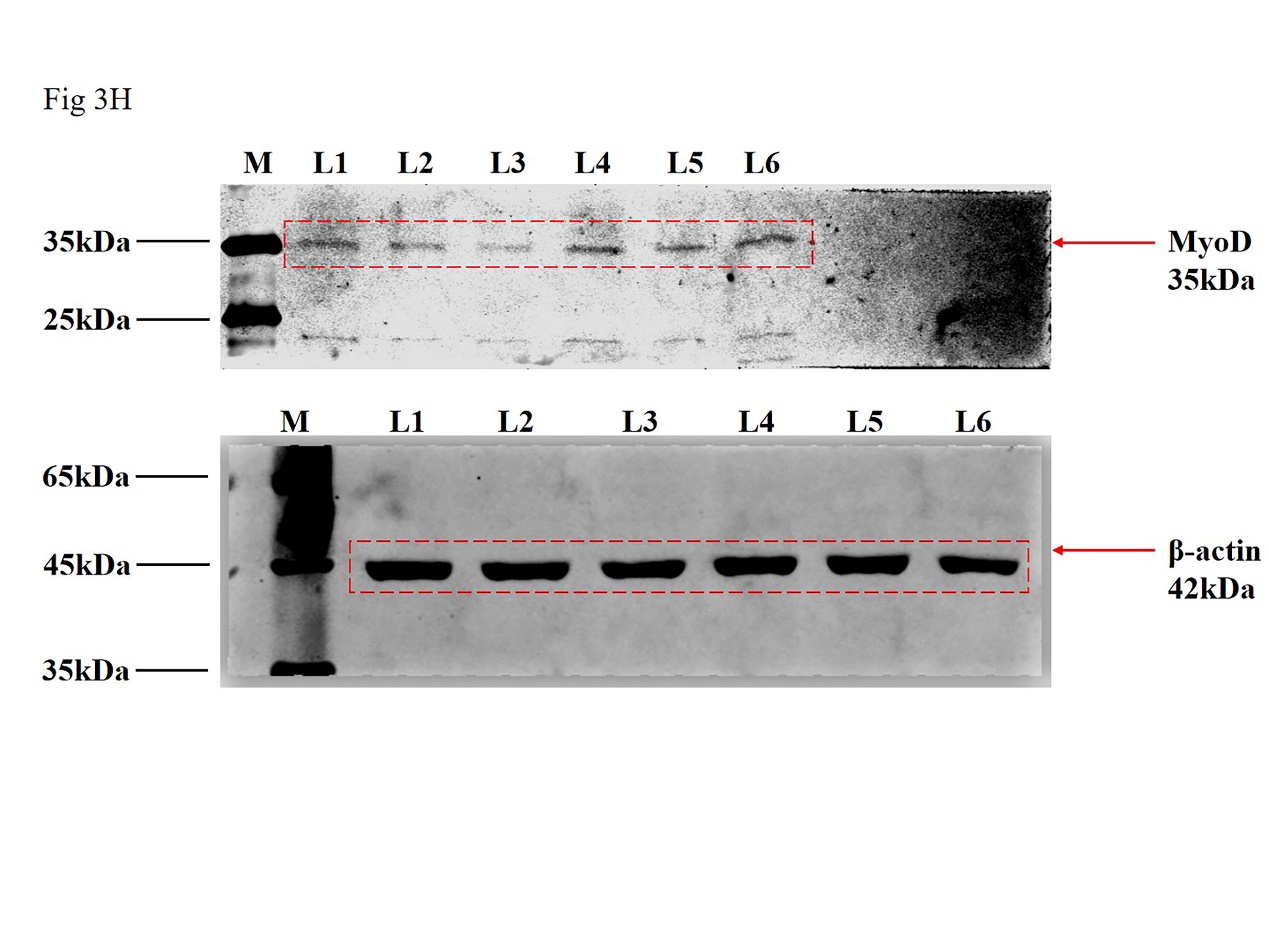


MyoG


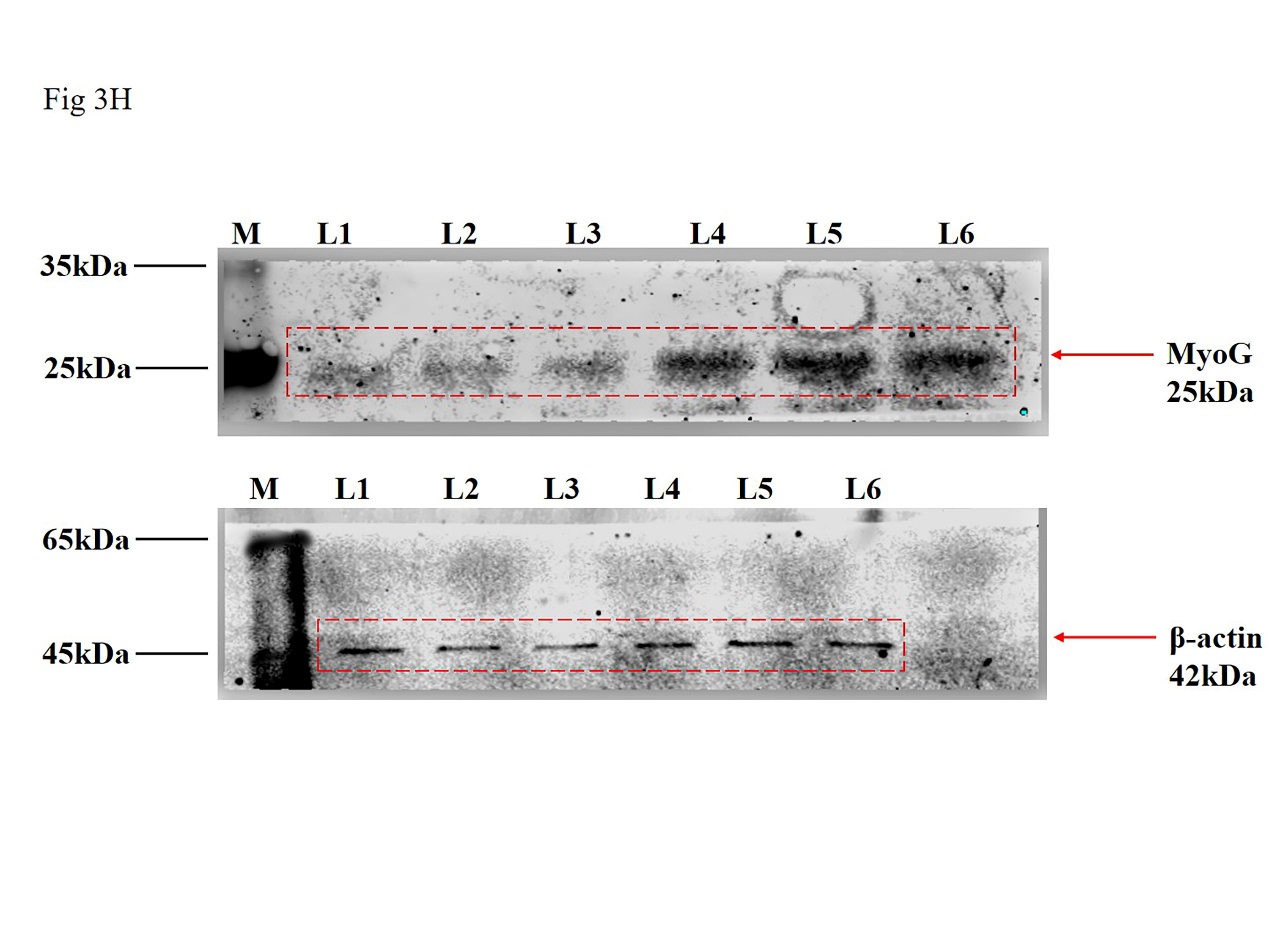


MHC


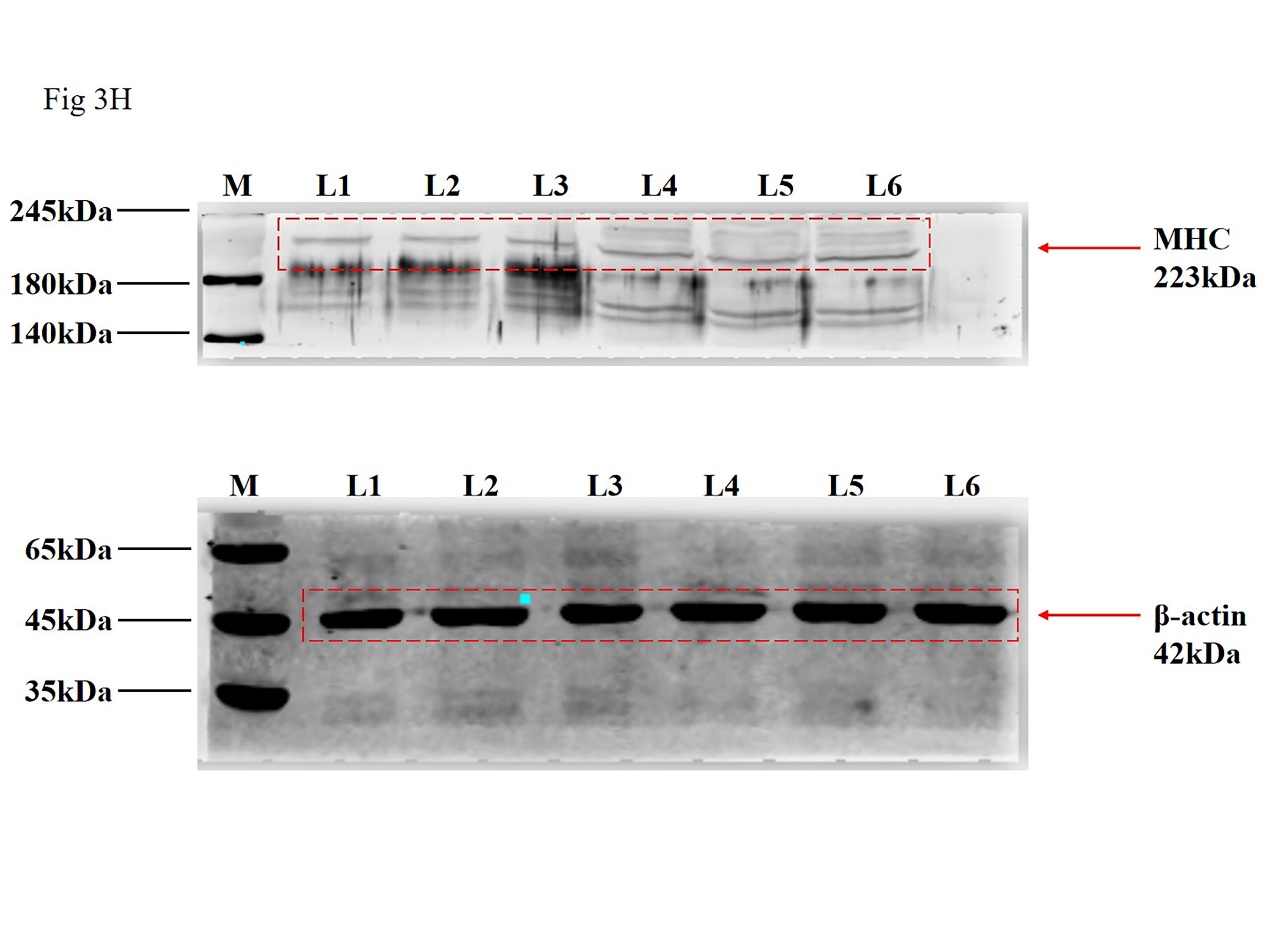


Figure.4D L1-L3: Control; L4-L6: RA

ID3


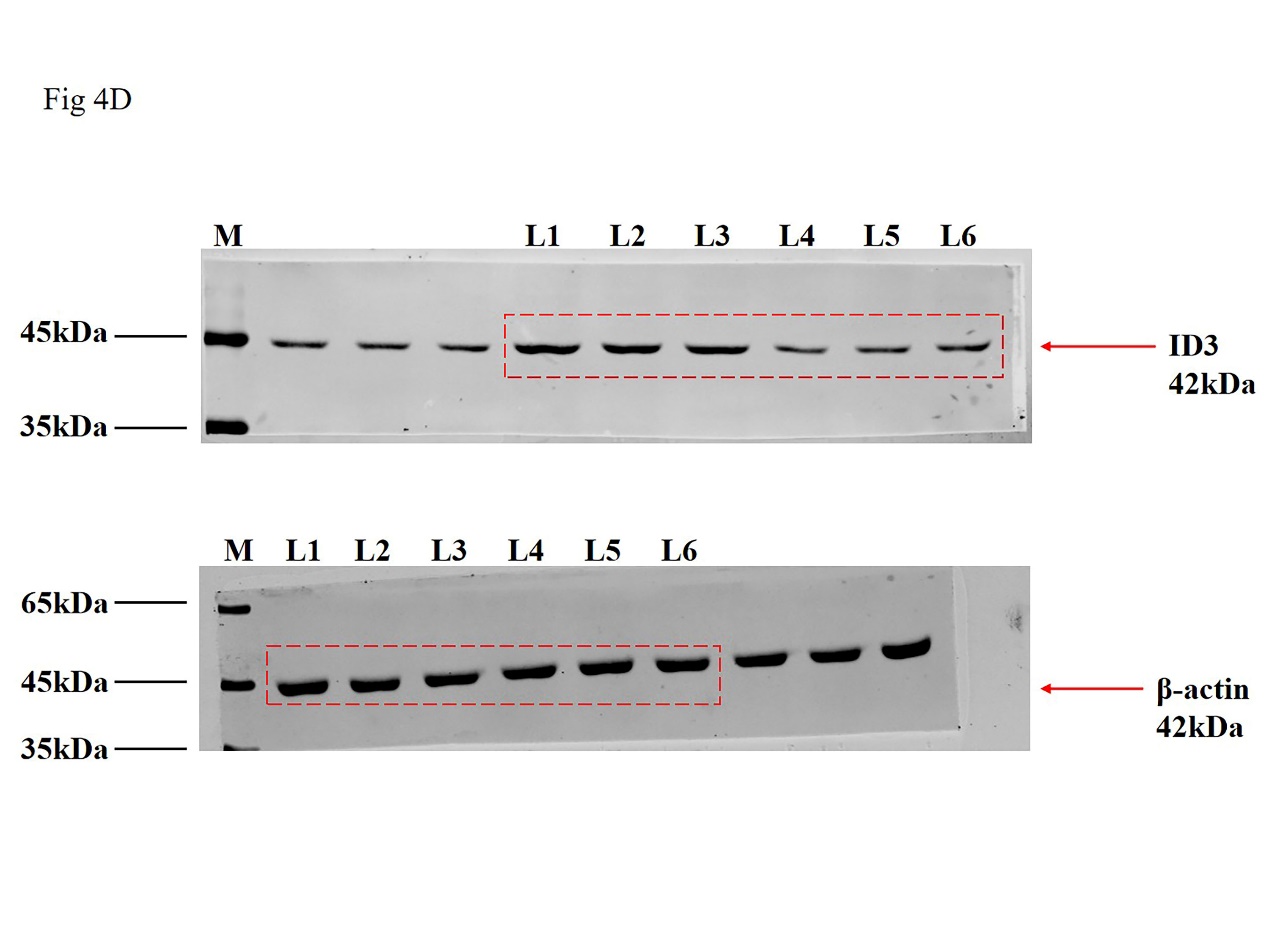


Figure.4F L1-L3: si-NC; L4-L6: si-BHLHE40.

ID3


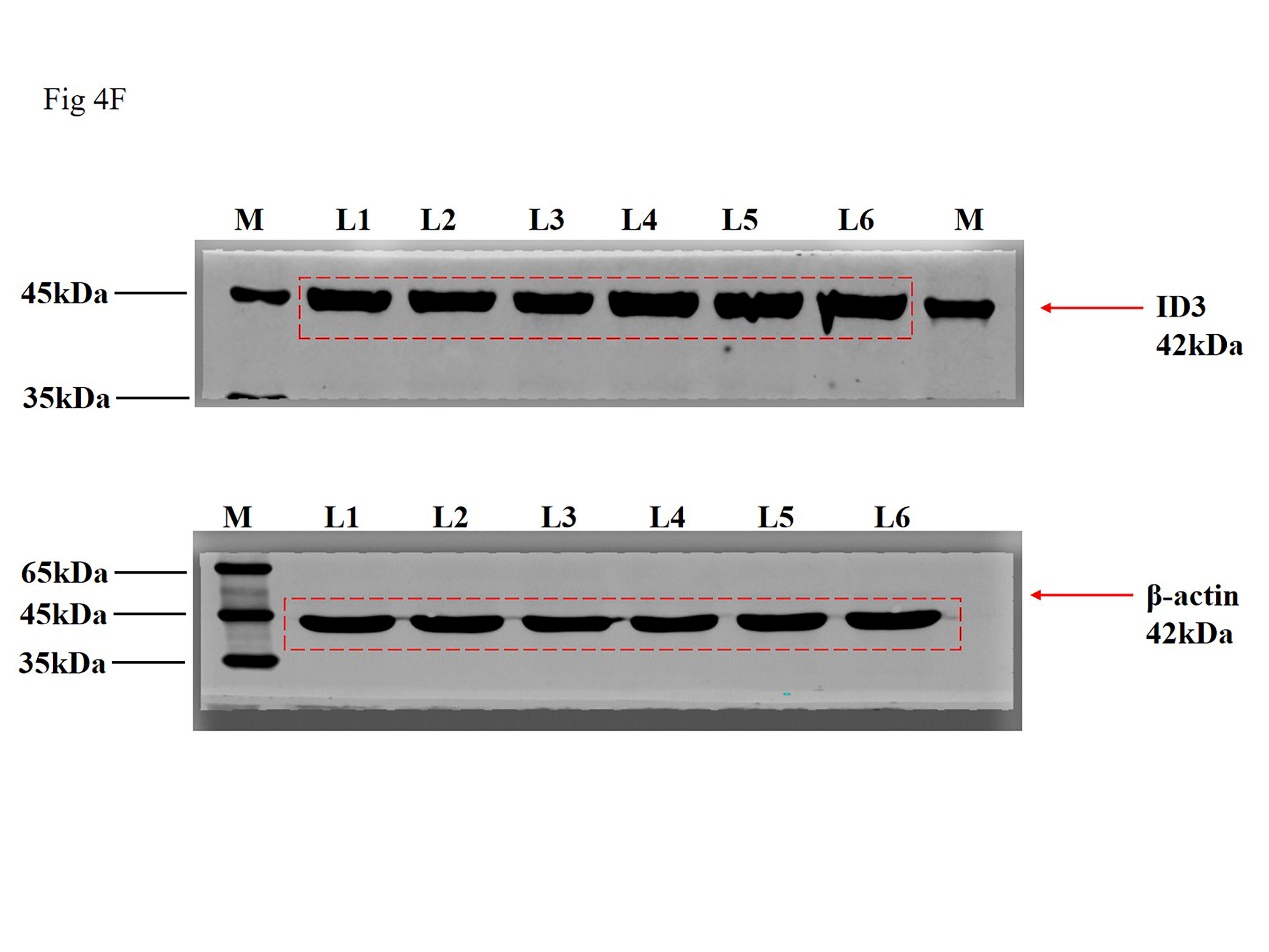


Figure.4G L1-L3: Vector; L4-L6: BHLHE40.

ID3


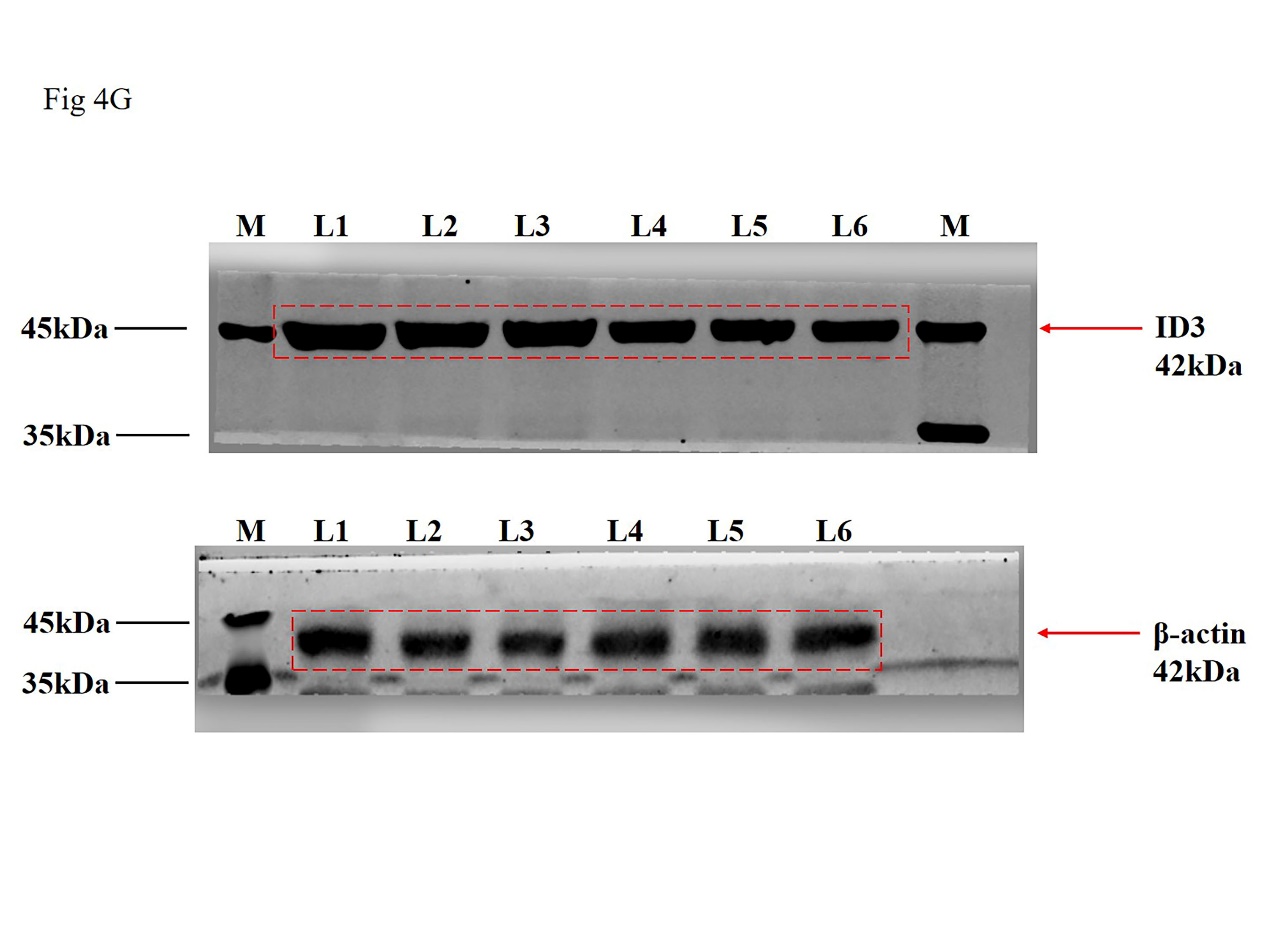


Figure.5C L1-L3: si-NC; L4-L6: si-ID3.

CDK4


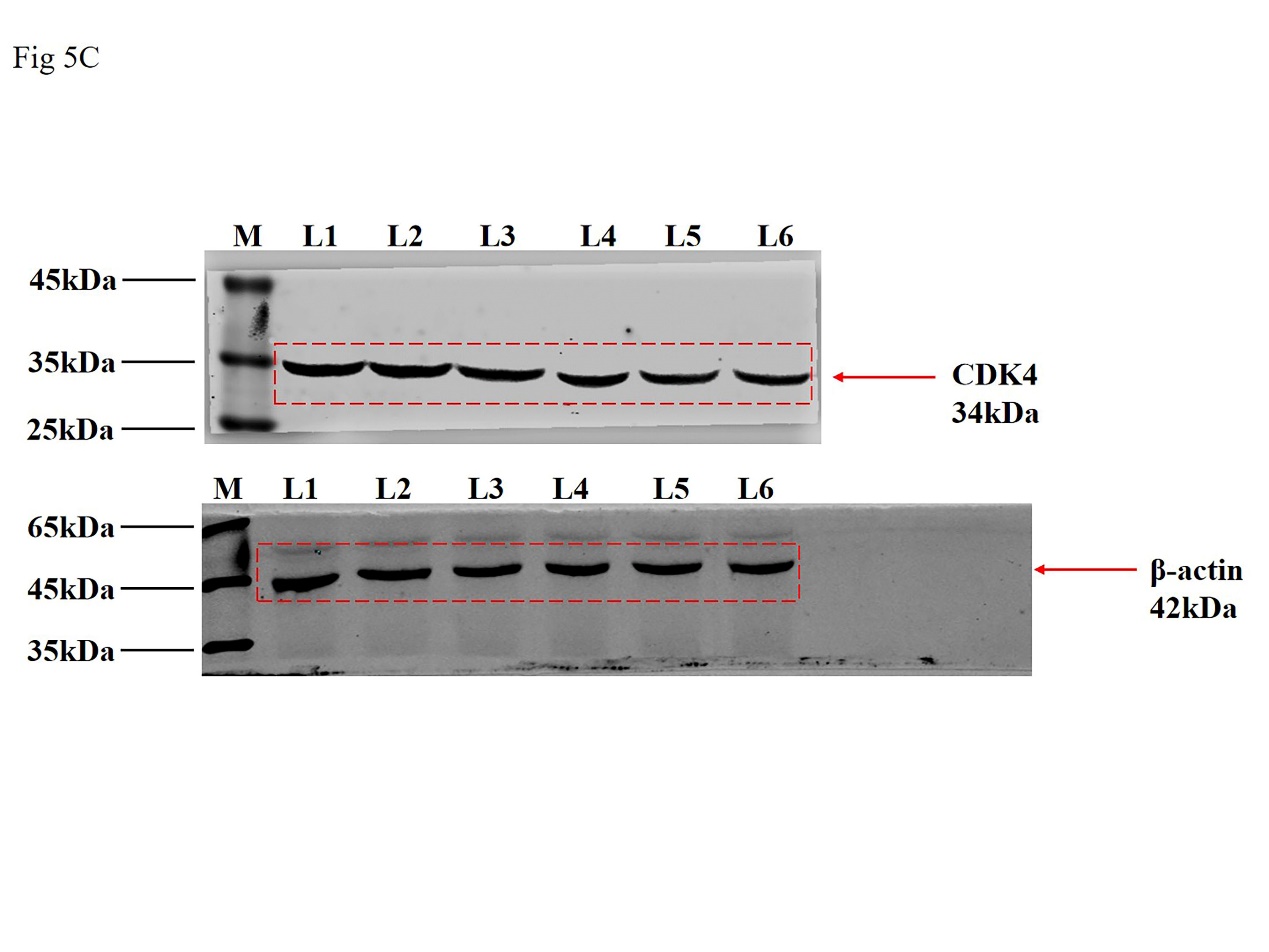


CyclinD1


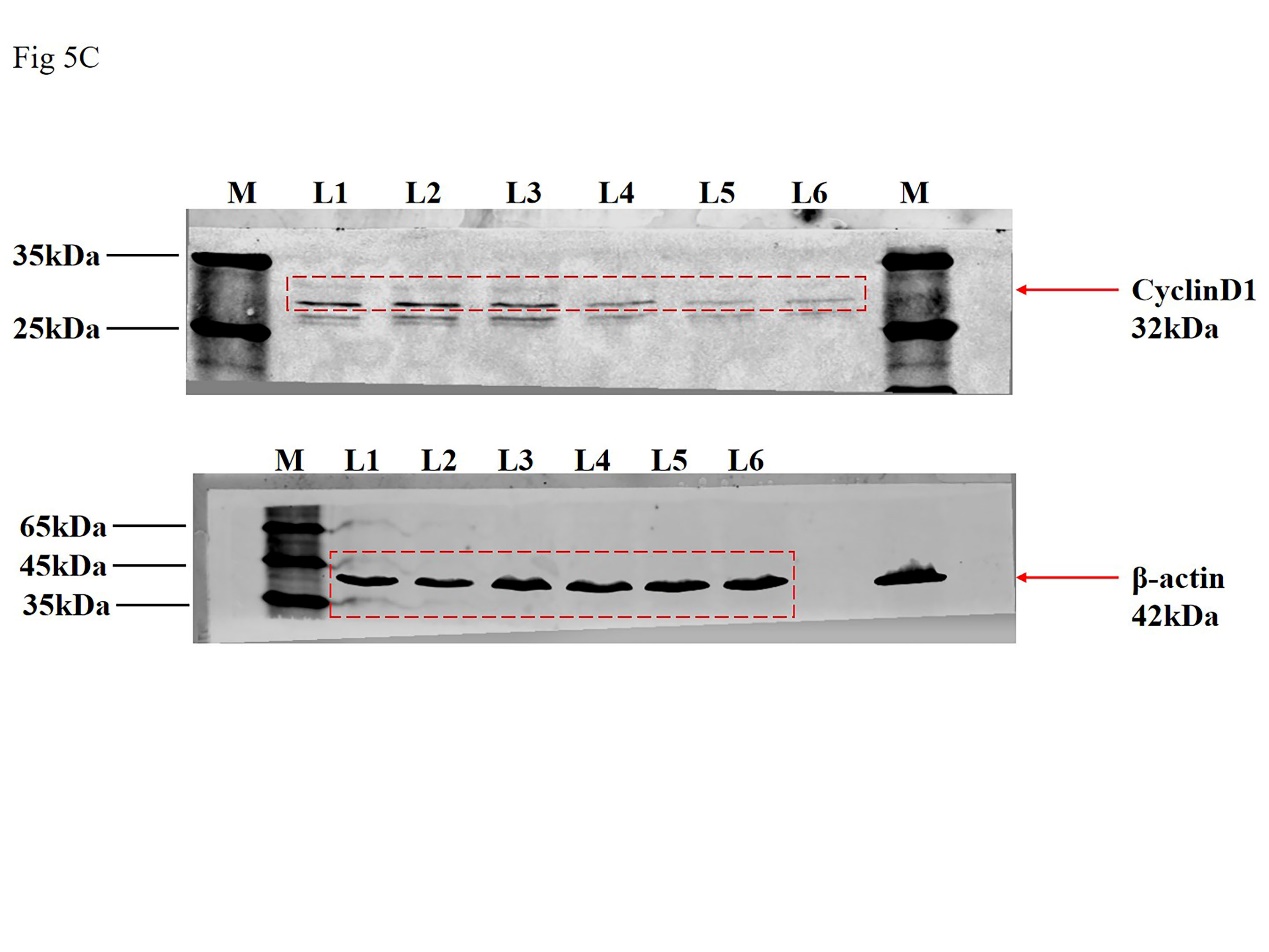


PCNA


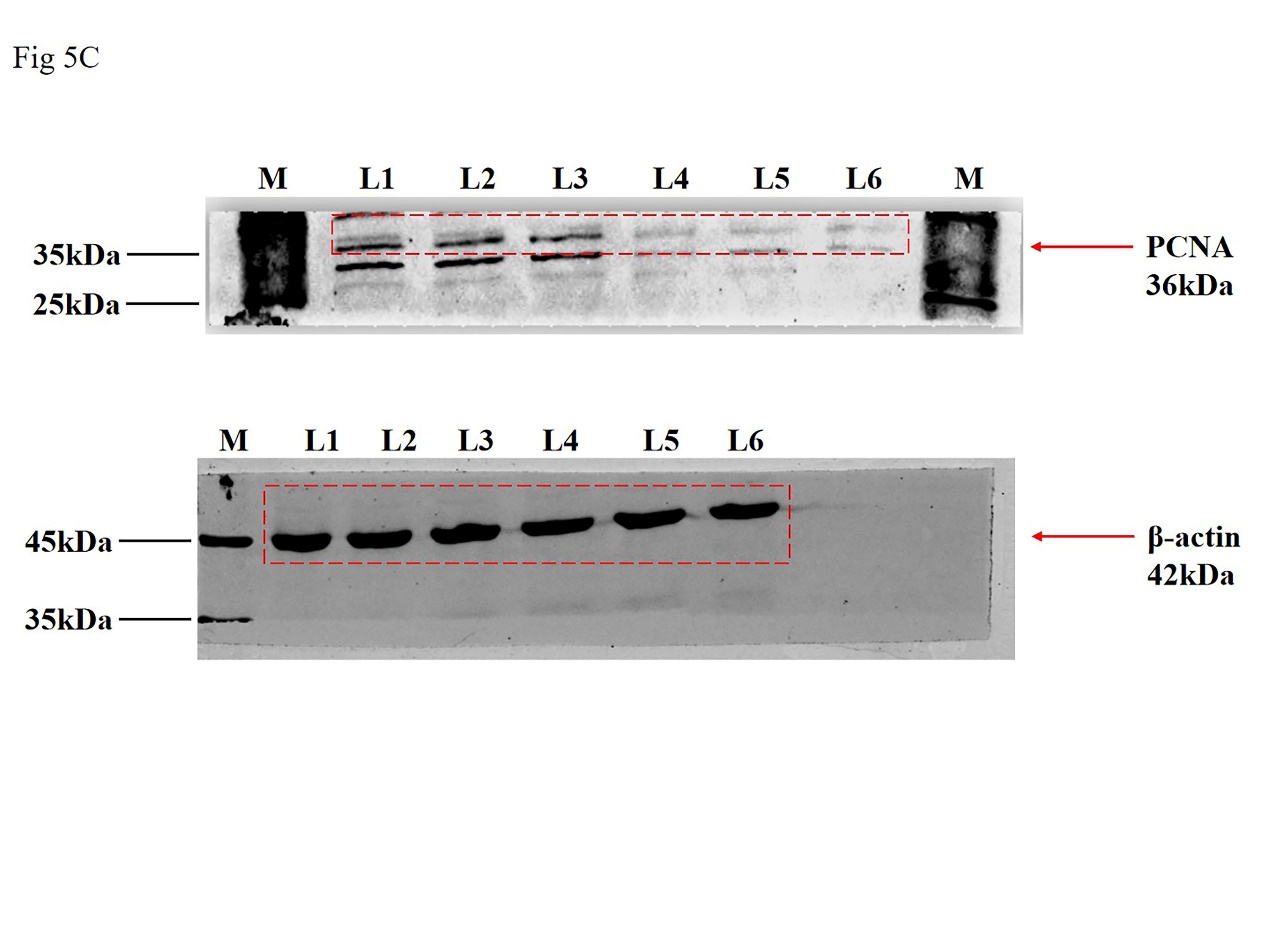


Figure.5F L1-L3: Vector; L4-L6: ID3.

CDK4


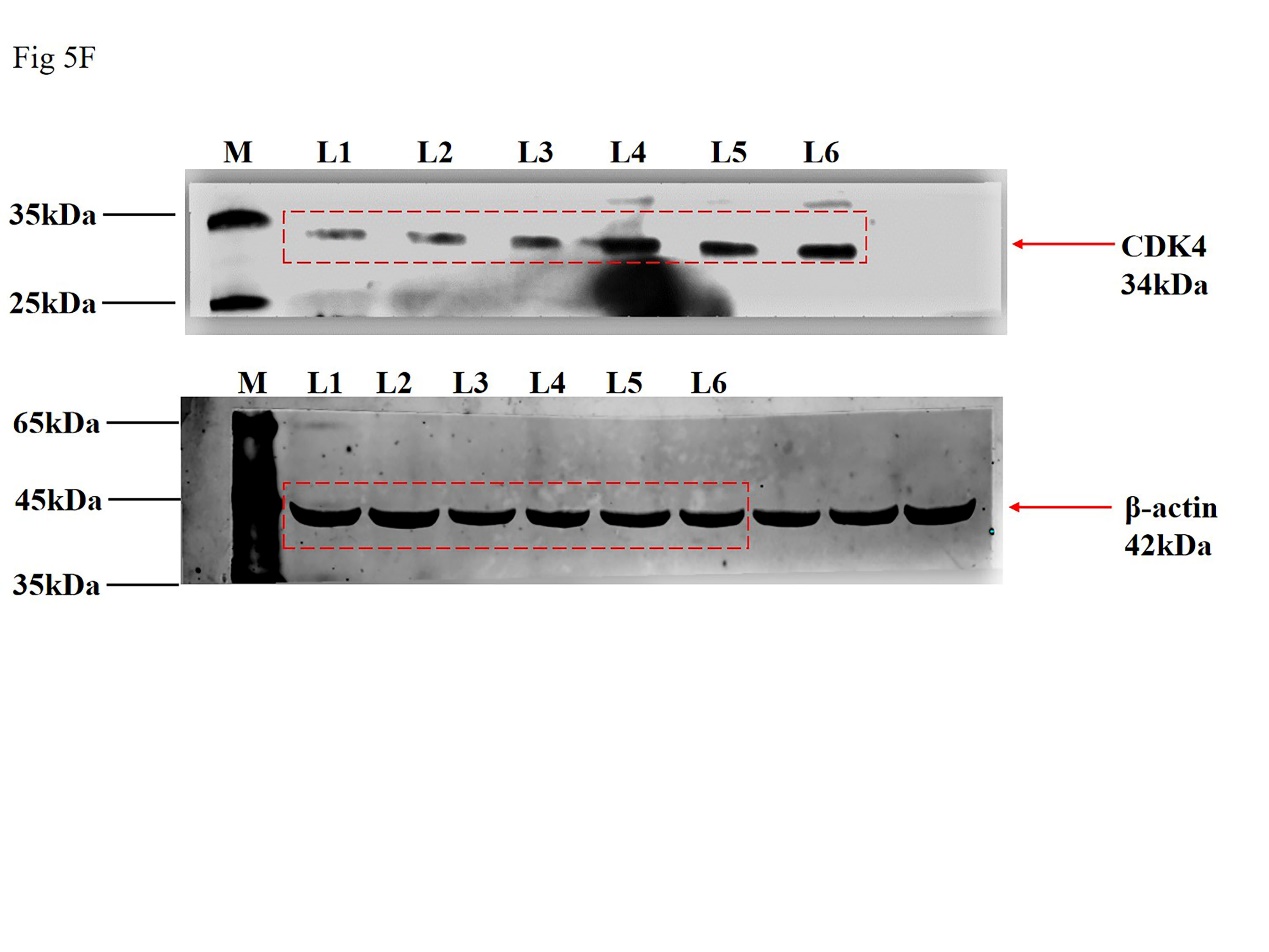


CyclinD1


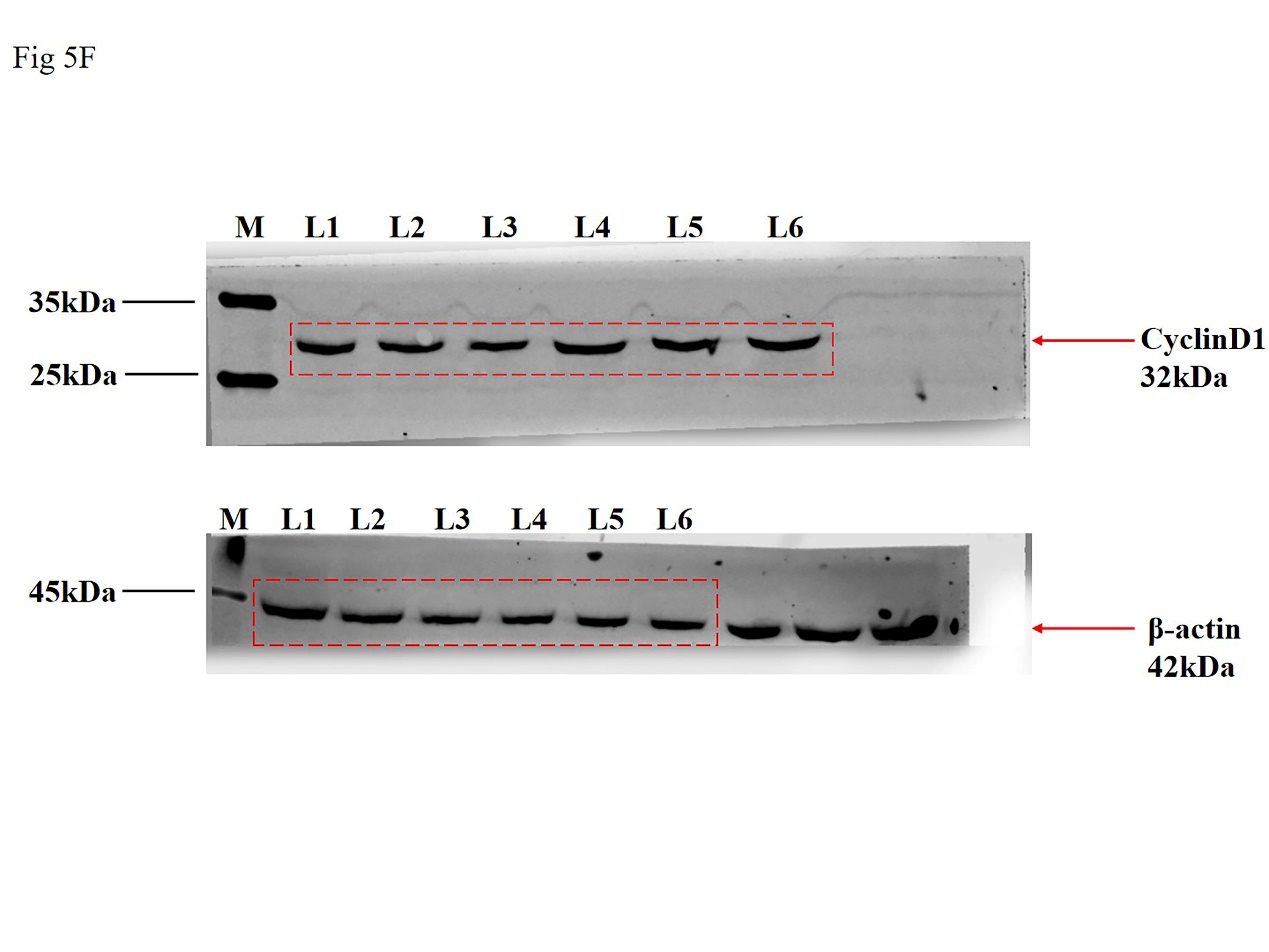


PCNA


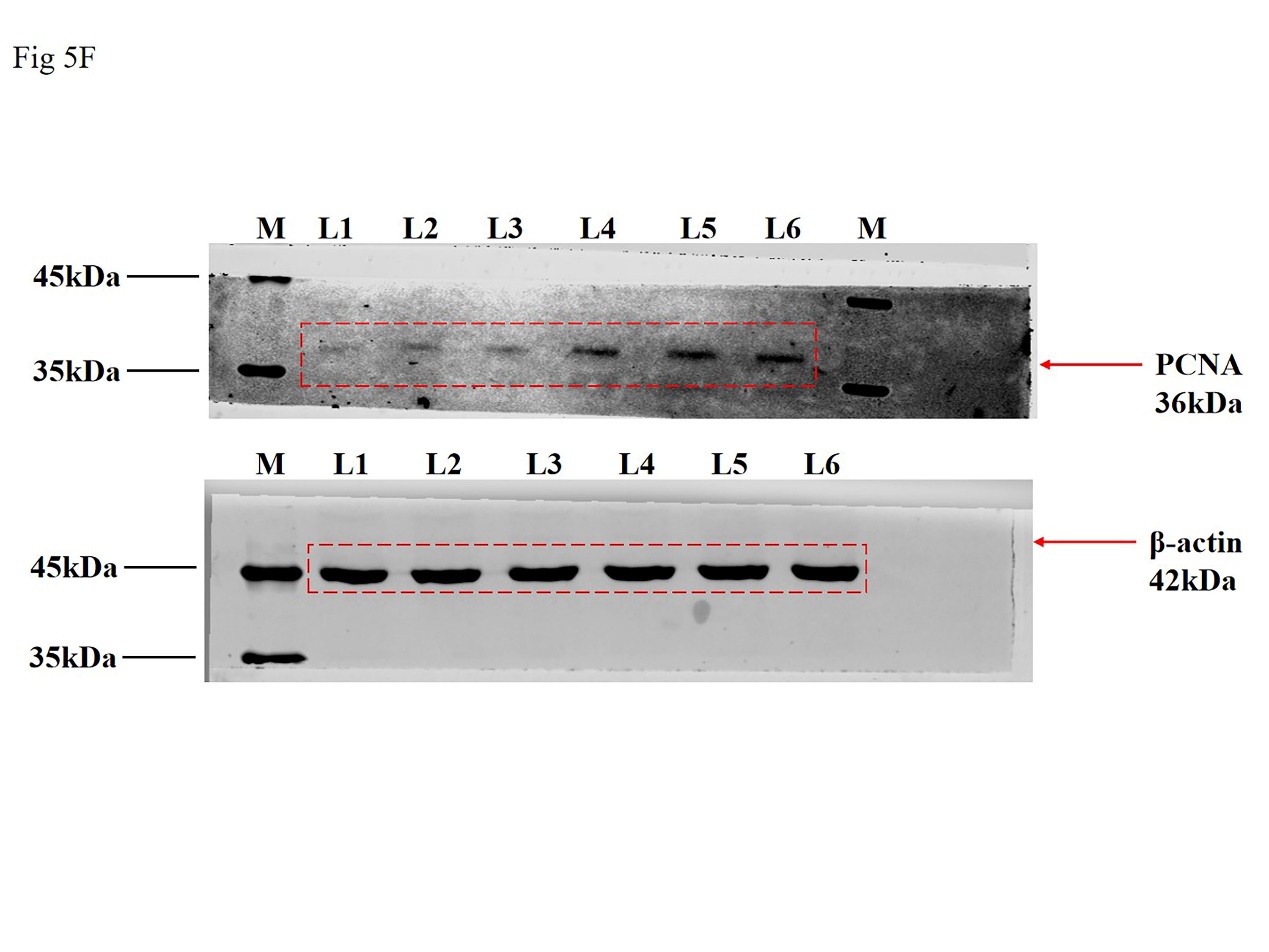


Figure.6D L1-L3: si-NC; L4-L6: si-ID3.

MyoD


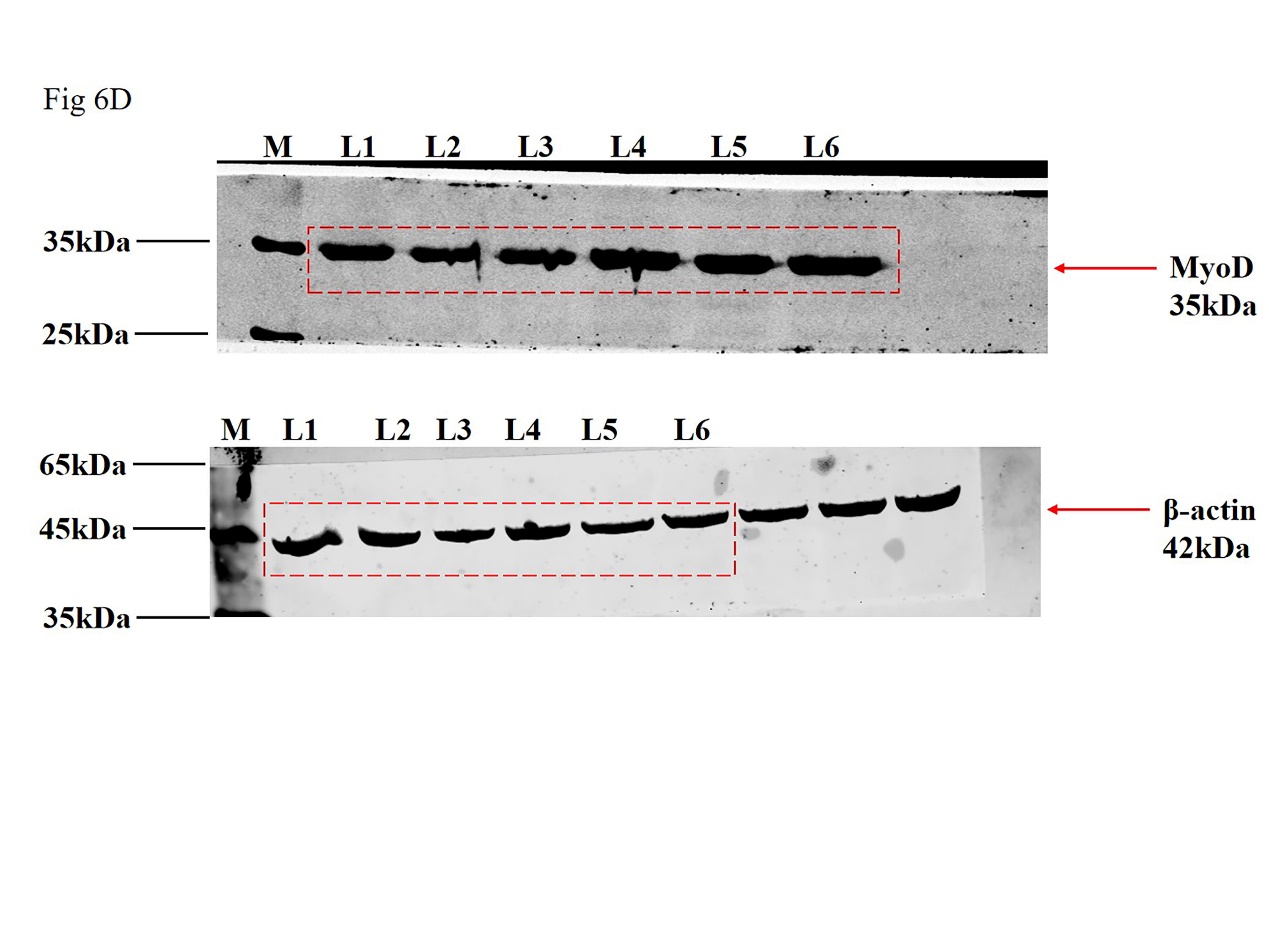


MyoG


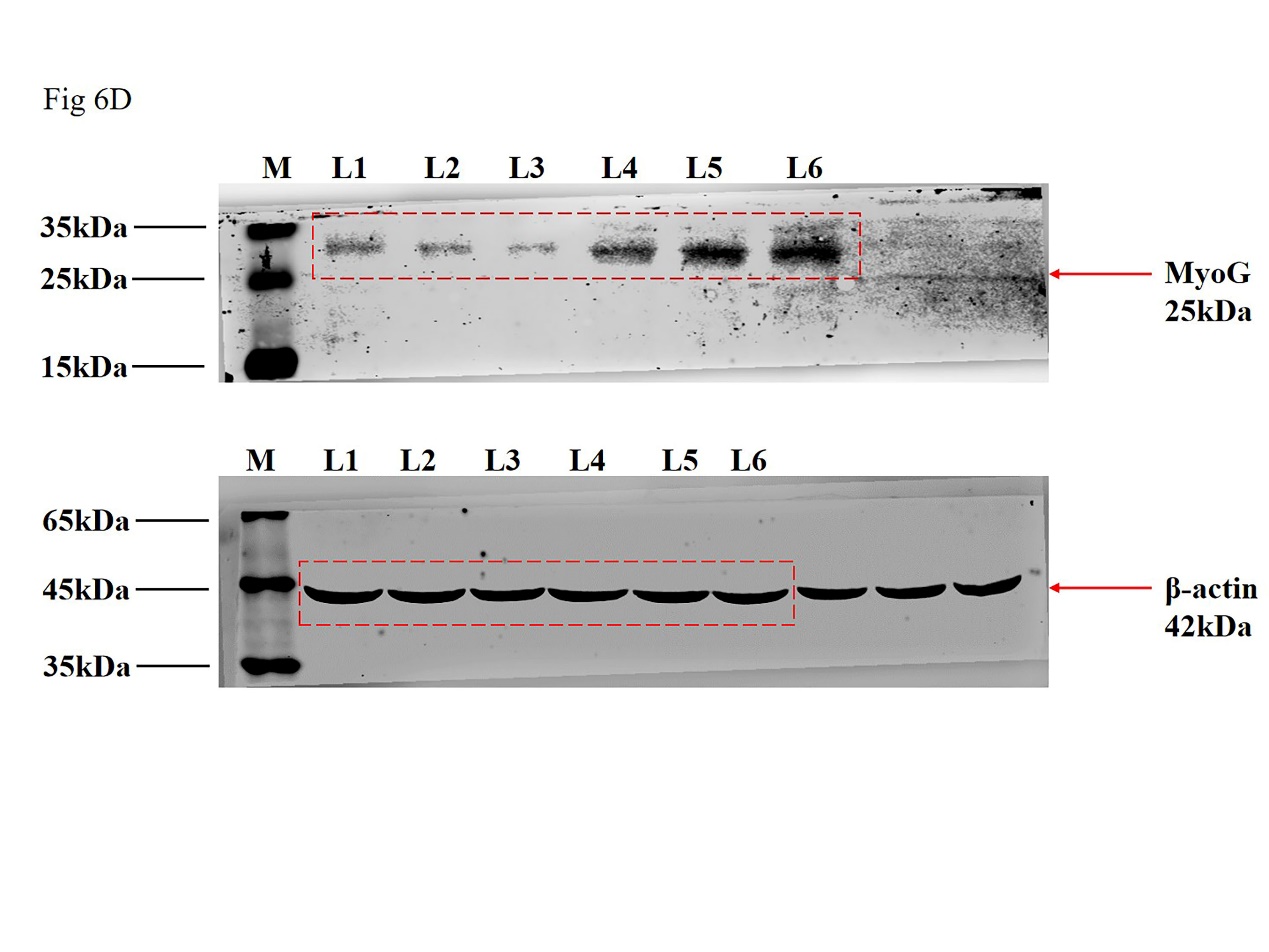


MHC


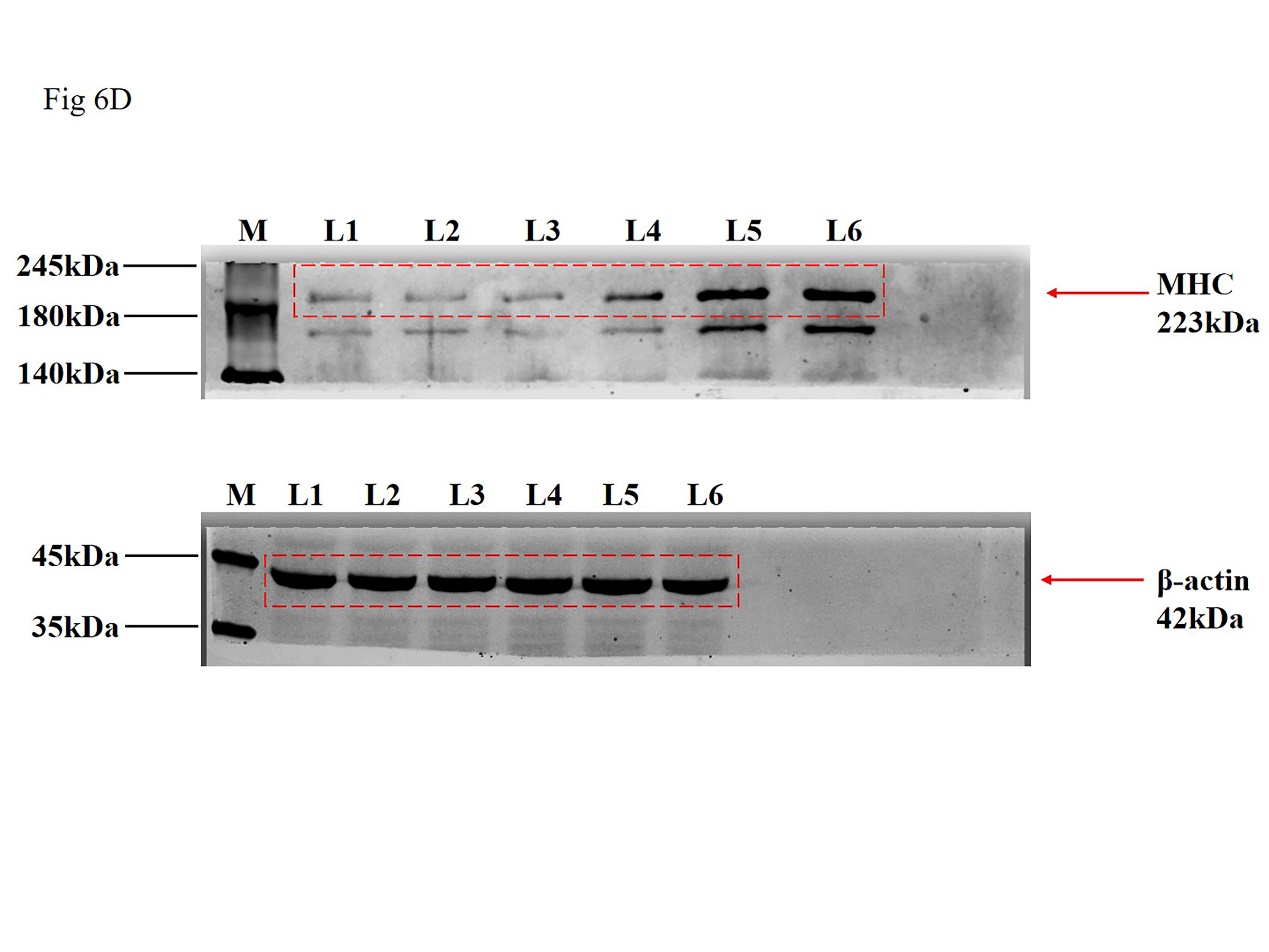


Figure.6H L1-L3: Vector; L4-L6: ID3.

MyoD


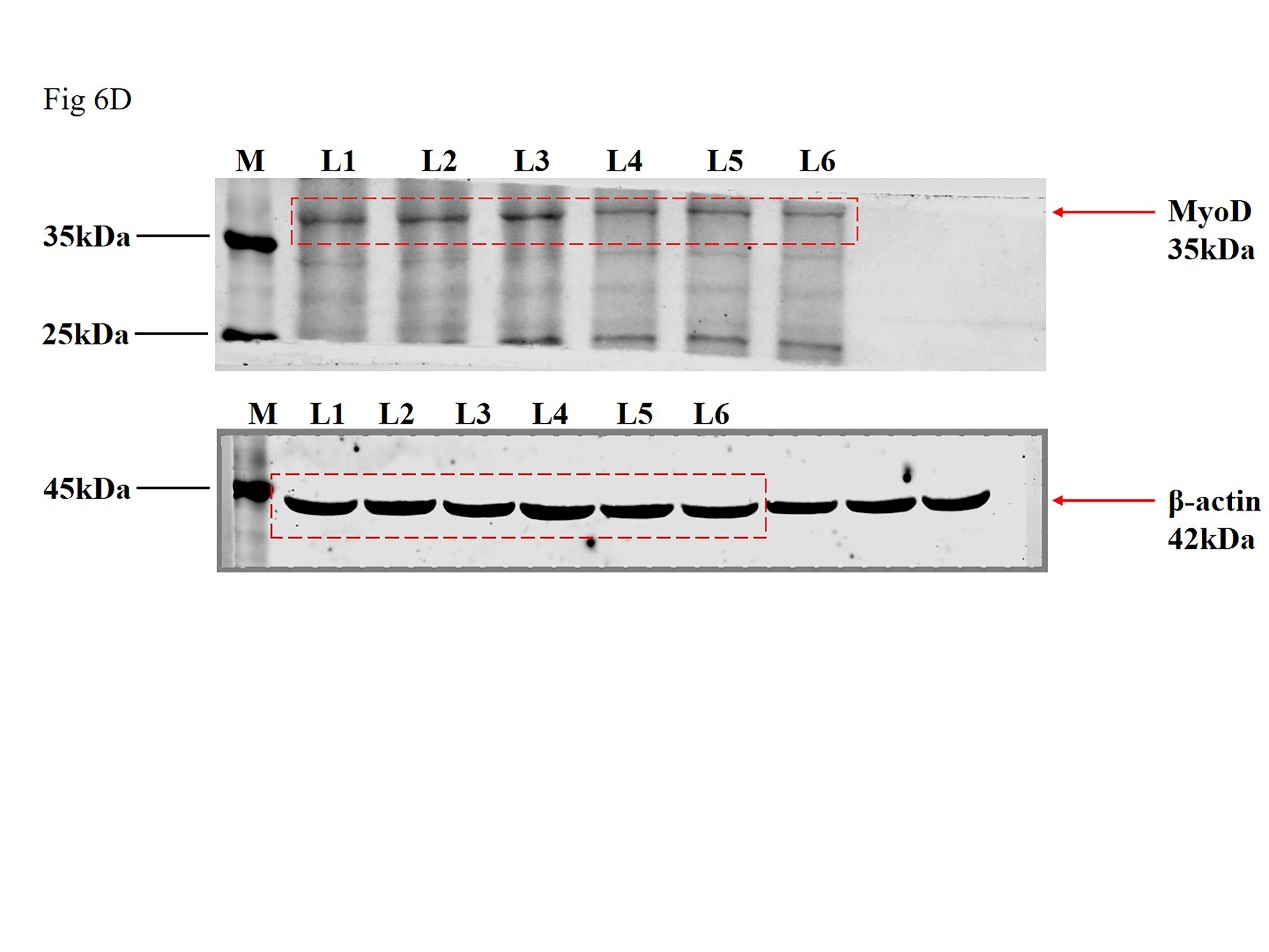


MyoG


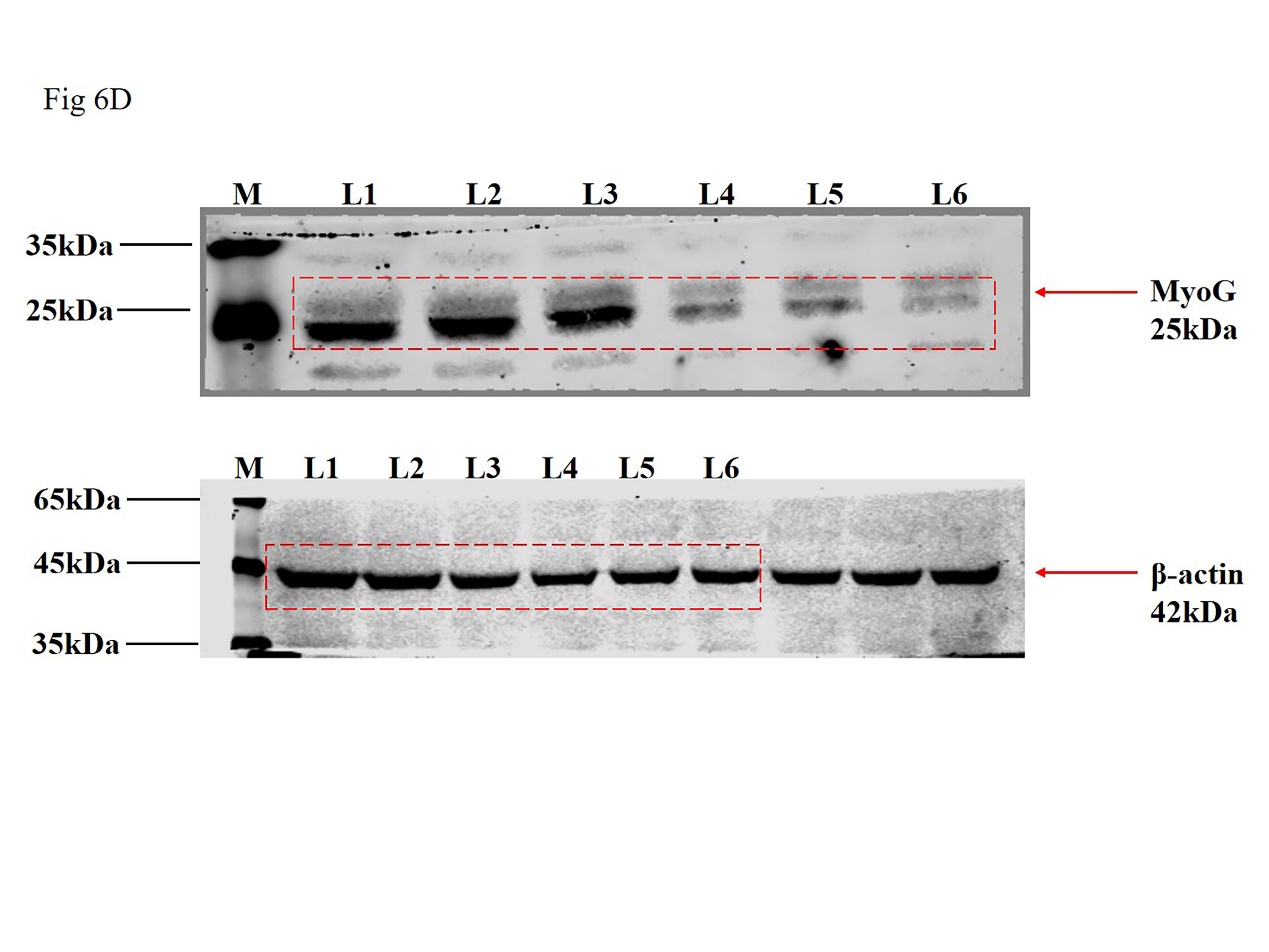


MHC


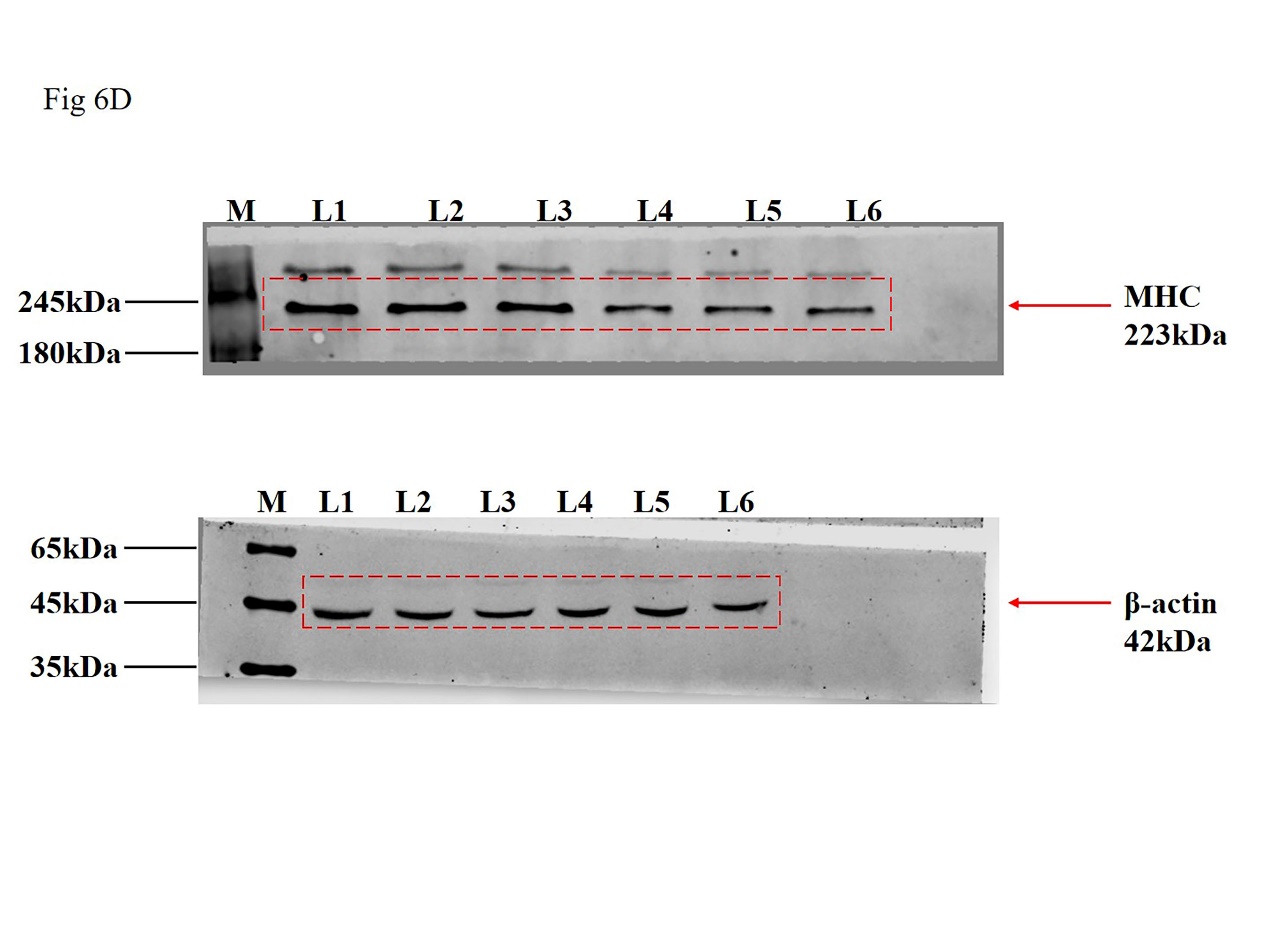

Supplement: Supplementary file 4 — Supplementary Material 4 [file 12864_2024_10161_MOESM4_ESM.docx]
